# Natural antioxidant products and nanomaterial-based delivery systems for the amelioration of diabetic retinopathy: mechanisms, applications, and translational perspectives

**DOI:** 10.3389/fimmu.2026.1911463

**Published:** 2026-09-09

**Authors:** Yulin Tao, Bingxu Zha, Fangpu Zhang, Wenrui Du, Siyu Lin, Xiang Gao, Weiwei Tang, Jie Gao, Zhengxuan Jiang

**Affiliations:** 1 Department of Ophthalmology, The Second Affiliated Hospital of Anhui Medical University, Hefei, Anhui, China; 2 Department of Clinical Medicine, The Second School of Clinical Medicine, Anhui Medical University, Hefei, Anhui, China

**Keywords:** anti-angiogenesis, diabetic retinopathy, drug delivery, nanomedicine, natural antioxidants, oxidative stress

## Abstract

Diabetic retinopathy (DR) is a leading global cause of vision loss, closely linked to hyperglycemia-induced oxidative stress, chronic inflammation, and pathological angiogenesis. Natural antioxidants exhibit great therapeutic potential against DR but are severely limited by poor solubility, low bioavailability, and insufficient ocular delivery. This review systematically summarizes the classification, pharmacological mechanisms, and therapeutic values of natural antioxidants in DR, and highlights advances in nanotechnology-based delivery systems to improve efficacy and clinical translation. We integrate preclinical and clinical studies focusing on key pathways including Nrf2/HO-1, NF-κB, NLRP3 inflammasome, and VEGF/HIF-1α. Flavonoids, polyphenols, carotenoids, and alkaloids exert multi-target effects by scavenging ROS, suppressing inflammation, and inhibiting pathological angiogenesis. Nanocarriers including lipid–polymer hybrid nanoparticles, self-nanoemulsifying drug delivery systems (SNEDDS), nanozymes, and lignin-based hydrogels significantly enhance solubility, corneal penetration, retinal targeting, and therapeutic efficacy. Representative nanoformulations effectively alleviate retinal oxidative damage, inflammation, and neovascularization. Natural antioxidants are promising DR candidates, and nanotechnology overcomes translational barriers. This review offers updated insights for developing natural product-based nanomedicines and proposes standardized formulations, biomarker-guided personalized therapy, and multi-target strategies to accelerate clinical translation.

## 1 Introduction

Diabetic retinopathy (DR) stands as one of the most prevalent and debilitating ocular complications arising from diabetes mellitus (DM), representing a leading cause of vision impairment and blindness worldwide. The global burden of diabetes continues to escalate, with projections indicating a significant rise in prevalence over the coming decades, thereby amplifying the incidence of associated microvascular complications such as DR (1). DR primarily results from progressive damage to the retinal microvasculature induced by chronic hyperglycemia, which triggers a cascade of pathological processes including oxidative stress, inflammation, and microvascular dysfunction. These mechanisms collectively contribute to retinal ischemia, neovascularization, and ultimately vision loss if left untreated (2, 3). The clinical manifestation of DR ranges from mild non-proliferative stages characterized by microaneurysms and retinal hemorrhages to advanced proliferative stages marked by neovascularization and vitreous hemorrhage (4, 5). Despite advances in diagnostic imaging and therapeutic interventions such as anti-vascular endothelial growth factor (VEGF) agents and laser photocoagulation, DR remains a significant public health challenge due to its asymptomatic early phases and the limitations of current treatments in reversing disease progression (5, 6).

**Figure 1:**
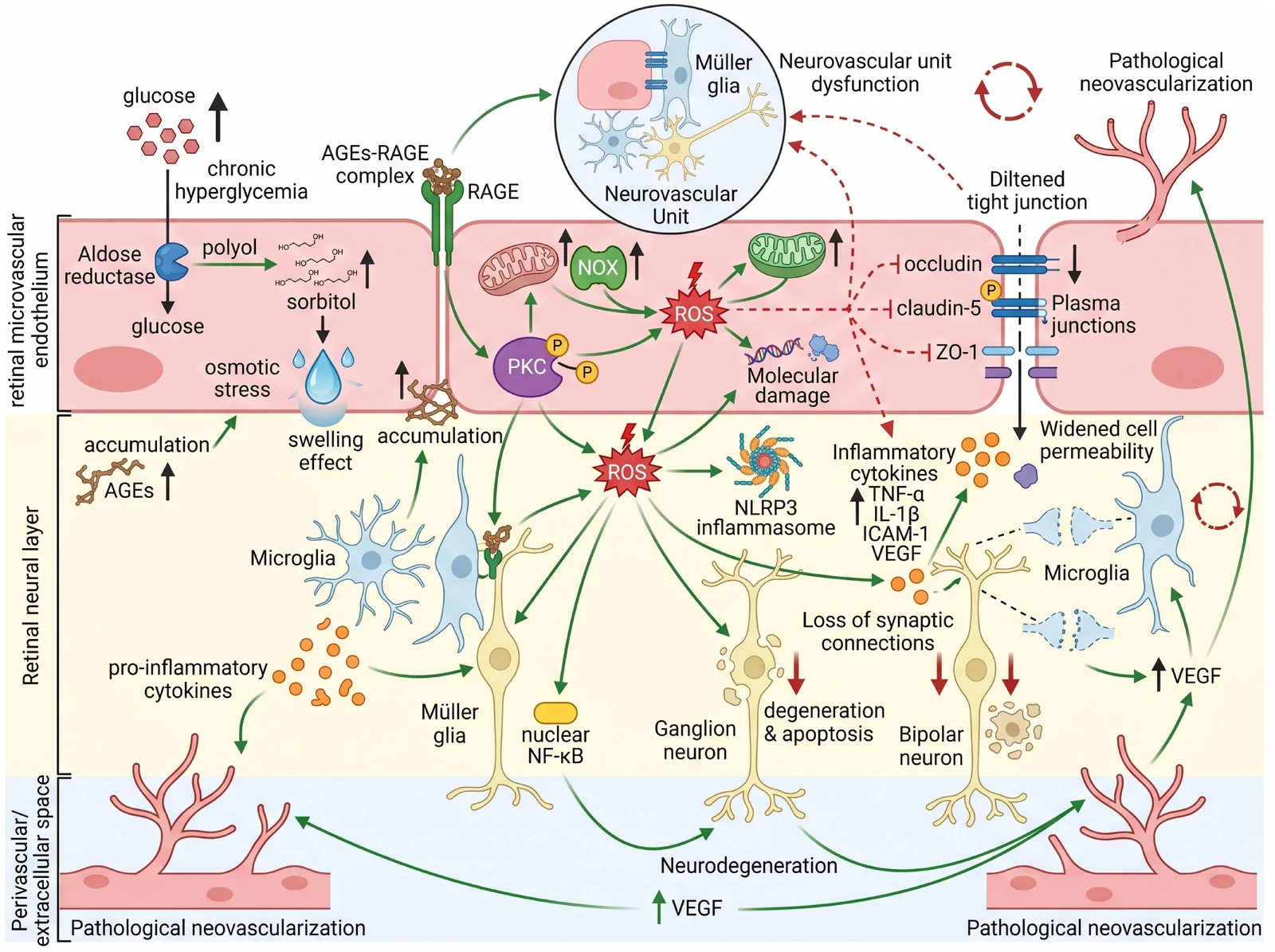
Schematic illustration of key pathological mechanisms in diabetic retinopathy. Chronic hyperglycemia triggers multiple pathways, including polyol, AGE-RAGE, PKC, and NOX, leading to excessive ROS production. This induces oxidative stress, inflammation, neurovascular unit dysfunction, blood-retinal barrier breakdown, neurodegeneration, and pathological neovascularization.

The pathogenesis of DR is multifactorial, with hyperglycemia-induced oxidative stress playing a central role. Elevated glucose levels enhance the generation of reactive oxygen species (ROS) through multiple biochemical pathways, including the polyol pathway, protein kinase C activation, formation of advanced glycation end products (AGEs), and the hexosamine biosynthetic pathway (**Figure 1**). This oxidative milieu leads to endothelial dysfunction, inflammation, and apoptosis within the retinal neurovascular unit, exacerbating microvascular damage (3, 7). Inflammatory signaling pathways, notably involving nuclear factor kappa B (NF-κB), contribute to the upregulation of pro-inflammatory cytokines and adhesion molecules, further perpetuating vascular injury and retinal degeneration (8, 9). Moreover, dysregulation of growth factors such as VEGF under hypoxic conditions promotes pathological neovascularization, a hallmark of proliferative DR (3, 10) (**Figure 1**). Recent molecular insights have also highlighted the involvement of microRNAs and metabolic alterations in DR progression, underscoring the complexity of its pathophysiology and the need for multifaceted therapeutic approaches (6, 11).

Given the intricate interplay of oxidative stress, inflammation, and vascular dysfunction in DR, there is growing interest in exploring natural antioxidant compounds as potential adjunctive therapies. Natural products, including polyphenols, flavonoids, carotenoids, and other phytochemicals, possess multifaceted biological activities such as antioxidant, anti-inflammatory, anti-angiogenic, and neuroprotective effects that may counteract the pathogenic mechanisms underlying DR (10, 12, 13). For instance, compounds like curcumin, alpha-lipoic acid (ALA), pterostilbene, and astaxanthin have demonstrated efficacy in preclinical models by reducing oxidative damage, modulating inflammatory pathways, and inhibiting VEGF-mediated neovascularization (14–17). These natural antioxidants may restore redox homeostasis, enhance endogenous defense systems such as the nuclear factor erythroid 2-related factor 2 (Nrf2) pathway, and improve retinal vascular and neuronal health (12, 18). Furthermore, advances in drug delivery systems, including nanotechnology, have improved the bioavailability and targeted delivery of these compounds to ocular tissues, enhancing their therapeutic potential (12, 19). Promising as the supporting experimental evidence may be, bench-to-bedside translation of natural antioxidant therapies for DR encounters substantial hurdles with respect to bioavailability, target specificity, and overall safety profiles. Comprehensive clinical trials are needed to validate their efficacy and optimize dosing regimens (13, 20). Additionally, integrating lifestyle modifications, including diet rich in antioxidant-containing fruits and vegetables, alongside conventional treatments may offer a holistic strategy to prevent or delay DR onset and progression (8). The multifactorial nature of DR pathogenesis, centered on oxidative stress and inflammation, positions natural antioxidant products as a compelling area of research. Their potential to modulate key pathogenic pathways offers hope for more effective, safe, and accessible interventions in the management of DR.

Despite the growing interest in natural antioxidants for DR management, the existing literature has largely addressed this field from fragmented perspectives. Several recent reviews have examined either natural products or nanotechnology-based delivery for DR, but a comprehensive integration of both aspects—spanning the full spectrum of natural antioxidant classes, their detailed molecular mechanisms, and the latest advances in nanomaterial-based delivery systems designed to overcome bioavailability and targeting barriers—remains lacking. For instance, the previous review focused on nanomaterial-enabled delivery of plant-derived bioactive metabolites but provided limited mechanistic detail (21). Another review offered a general overview of natural products in DR but dedicated minimal attention to nanotechnology integration (20). The review published in Antioxidants concentrated exclusively on polyphenols and oxidative stress pathways, without comprehensive coverage of other antioxidant classes or detailed nanomaterial delivery strategies (22). While these contributions have advanced the field, a review that systematically integrates the full spectrum of natural antioxidant classes, their detailed molecular mechanisms, and the latest nanotechnology-based delivery systems—all within a clinical translation framework—remains absent.

Our review addresses this critical gap by providing: (1) a systematic classification of natural antioxidants across all major phytochemical categories (flavonoids, polyphenols, carotenoids, alkaloids, terpenoids, and phenylpropanoids) with detailed mechanistic analyses targeting key pathways including Nrf2/HO-1, NF-κB, NLRP3 inflammasome, and VEGF/HIF-1α; (2) comprehensive tabular summaries of molecular targets and therapeutic mechanisms for over 40 natural compounds; (3) a dedicated overview of nanomaterial-based delivery systems, including lipid-polymer hybrid nanoparticles, self-nanoemulsifying drug delivery systems (SNEDDS), nanozymes, lignin-based hydrogels, and emerging platforms; (4) a critical examination of clinical trial results and translational challenges; and (5) forward-looking recommendations for biomarker-guided personalized therapy and multi-target strategies. By integrating the mechanistic actions of natural antioxidants with nanotechnology-based solutions to overcome bioavailability and targeting barriers, this review provides a translational roadmap for developing effective natural product-based therapies for DR.

The distinct advantages of our review are fourfold. First, the breadth of coverage—encompassing all major natural antioxidant classes with detailed mechanistic pathways—is not available in any single existing publication. Second, the comprehensive tabular resources (**Tables 1**, **2**) serve as definitive reference tools for researchers. Third, the integrated focus on both mechanisms and nanomaterial delivery provides a complete translational perspective. Fourth, the critical analysis of clinical challenges and personalized therapy approaches offers practical guidance for future research and clinical application. Together, these features position our review as a valuable resource for ophthalmologists, pharmacologists, and materials scientists working on DR therapeutics.

**Table 1:** Functional targets and molecular mechanisms of natural antioxidants in the treatment of diabetic retinopathy.

Natural antioxidant product (category)	Target molecule or pathway	Mechanism of action	Study type	References
1. Polyphenols				
1.1 Flavonoids*				
Quercetin	MAPK, ERK1/2, NF-κB, VEGF, ROS	Activates antioxidant pathways, inhibits inflammation and angiogenesis, reduces oxidative stress and apoptosis.	Animal/Cell	(126, 127)
Catechin/EGCG	VEGF, HIF-1α, NF-κB, MAPK/ERK-VEGF, MMP9, ROS/TXNIP/NLRP3, NMDA, H_2_O_2_	Inhibits VEGF expression, alleviates inflammatory response, protects retinal ganglion cells and vascular endothelial cells.	Animal/Cell	(128–131)
Kaempferol	PI3K/AKT, NF-κB, Nrf2, VEGF, PGF, Src-AKT1-Erk1/2, TSP-1, ADAMTS-1, VASH1	Exerts anti-inflammatory and antioxidant effects by activating PI3K/AKT and Nrf2 pathways and inhibiting NF-κB, protecting the blood-retinal barrier.	Animal/Cell	(132–136)
Luteolin	NF-κB, MAPK, VEGF, PPARγ, GLUT, NLRP/NOX4, SIRT1/P53, SQSTM1/BNIP3L, Nrf2/Keap1, SIRT3/AMPK	Inhibits high glucose-induced release of inflammatory factors and VEGF expression, reducing retinal vascular leakage.	Cell	(49, 137–141)
Apigenin	PTEN/PI3K/AKT, CBP, HDAC3	Activates endogenous antioxidant defense system while inhibiting inflammatory pathways, protecting retinal cells from high glucose damage.	Cell/Animal	(142, 143)
Scutellarin	NLRP3, VEGF/ERK/FAK/Src, HIF-1α,	Scutellarin exerts therapeutic effects on diabetic retinopathy through multiple mechanisms, including inhibiting NLRP3 inflammasome activation, suppressing RGC pyroptosis, downregulating the VEGF/ERK/FAK/Src and ROS/HIF-1α/VEGF signaling pathways to reduce angiogenesis.	Cell/Animal	(144–149)
Dihydromyricetin	Poldip2/Nox4/H_2_O_2_, SOD, GSH	Dihydromyricetin can alleviate diabetic retinopathy and high glucose-induced damage in retinal pigment epithelial cells by downregulating the Poldip2-Nox4-H_2_O_2_ pathway and miR-34a expression, thereby reducing oxidative stress and cell apoptosis.	Animal/Cell	(61, 150)
Baicalein	GFAP, VEGF, HIF-1α, TNF, PI3K-AKT, MAPK, PPARG, ALB	Baicalein exerts protective effects against diabetic retinopathy by attenuating inflammatory responses in rodent models and regulating ferroptosis as its underlying therapeutic mechanism.	Cell/Animal	(151, 152)
Shikonin	BAX, COX-2, iNOS, PKM2	Shikonin exerts beneficial effects on early-stage diabetic retinopathy primarily through its anti-inflammatory properties.	Animal/Cell	(153, 154)
Hesperidin	MAPK, JNK, DOT1L, FASN, SREBF1, ROS	Hesperidin alleviates diabetic retinopathy by reducing oxidative stress, apoptosis and VEGF expression.	Animal/Cell	(155–158)
Blueberry Anthocyanin Extracts (BAEs)	GLP-1R/AKT/ GSK3β	Protect Retinal and Retinal Pigment Epithelium Function from High-Glucose-Induced Apoptosis by Activating GLP-1R/AKT Signaling.	Animal/Cell	(159)
Puerarin	STAT3, IL-1β, NADPH, iNOS, AGEs/RAGE	Puerarin protects against diabetic retinal injury by inhibiting oxidative stress, reducing apoptosis of retinal cells, and suppressing inflammation and STAT3 expression.	Animal/Cell	(160–164)
Naringenin	Bax, Bcl2, VEGF, GTPCH1/Enos, ROS	Naringenin attenuates oxidative stress, apoptosis, and inflammation, upregulates GTPCH1/eNOS to protect retinal endothelial cells.	Cell/Clinical/Animal	(45, 165, 166)
1.2 Other polyphenols				
Resveratrol	CDKN2A, SIRT1/PGC-1α, Nrf2/HO-1, NF-κB, HMGB1, HIF-1α, HMOX1, HLA-A, Nrf2/GPx4/PTGS2, VEGF, AGEs	Resveratrol protects against diabetic retinopathy by activating Nrf2 signaling, inhibiting oxidative stress, inflammation and ferroptosis via SIRT1/HMGB1 and Nrf2/GPx4/PTGS2 pathways, while exerting immunomodulatory, anti-apoptotic effects and promoting autophagy.	Animal/Cell	(167–172)
Curcumin	ROS-PI3K/AKT/mTOR, Nrf2/HO-1, ERK1/2	Curcumin alleviates diabetic retinal injury by reducing oxidative stress, maintaining Nrf2 pathway homeostasis, activating Nrf2/HO-1 via ERK1/2, and suppressing inflammatory injury through the ROS-PI3K/AKT/mTOR pathway.	Animal/Cell	(42, 173, 174)
Green Tea Polyphenols	VEGF, MMP-9, NF-κB, Nrf2	(Represented by EGCG) Comprehensive antioxidant, anti-inflammatory, and anti-angiogenic effects, improving retinal microcirculation.	Animal/Clinical	(130, 131, 175)
Gallic Acid	AGEs/RAGE	Activates antioxidant defense system and inhibits AGEs-induced cytotoxicity.	Animal/Cell	(176–178)
Ellagic Acid	Nrf2/HO-1, AGEs	Inhibits high glucose-induced VEGF upregulation and activation of MAPK/NF-κB inflammatory pathways.	Cell/Animal	(179, 180)
Chlorogenic acid	TNFR1, TNFα, NFκB, VCAM1, ICAM1	Chlorogenic acid targets TNFR1 to block TNFα/NFκB signaling, reduces VCAM1/ICAM1 and inflammation, thereby mitigating BRB breakdown and leukocyte adhesion in diabetic retinopathy.	Animal/Cell	(181)
Rosmarinic acid	VEGF, RACK1, PKC, ROS	Rosmarinic acid and its intravitreal implants exert antiangiogenic effects to reduce neovascularization.	Animal/Cell	(182)
2. Carotenoids				
Lutein/Zeaxanthin	ROS, H_2_O_2_, GSTP1	As major pigments in the macular region, directly quench ROS and mitigate photo-oxidative damage.	Animal/Clinical	(87, 183)
Lycopene	ROS, NLRP3, VEGF, VE-cadherin	Potent singlet oxygen quencher, inhibits retinal cell apoptosis, inflammation, and angiogenesis.	Animal/Cell	(87, 184, 185)
β-Carotene	ROS, Antioxidant Enzyme Activity	Precursor of vitamin A, acts by scavenging free radicals and enhancing total antioxidant capacity.	Animal	(87, 186)
Astaxanthin	VEGF, ROS, NADPH, HIF1α, XBP1, Aldose reductase	Astaxanthin alleviates diabetic retinopathy by suppressing oxidative stress, cell apoptosis and aldose reductase activity in diabetic models.	Animal/Clinical	(46, 187–189)
3. Alkaloids and other nitrogen-containing compounds				
Berberine	AMPK, HIF-1α/VEGF/NF-κB, AKT/mTOR, GABA-α, NLRP3	Berberine ameliorates diabetic retinopathy by inhibiting HIF-1α/VEGF/NF-κB and NLRP3 inflammasome, activating GABA-α receptor, and regulating AMPK/mTOR-related autophagy and apoptosis.	Animal/Cell	(190–194)
Berberis dictyophylla F.	HIF-1α/VEGF/DLL-4/Notch-1	Inhibits angiogenesis and apoptosis of diabetic retinopathy via suppressing HIF-1α/VEGF/DLL-4/Notch-1 pathway.	Animal	(195)
Piperine	HIF-1α/VEGF, PEDF	Piperine exert protective effects on the retina of mice with diabetes via regulating the pro-antiangiogenic homeostasis composed of HIF-1/VEGFA and PEDF.	Animal/Clinical	(74, 196)
α-Lipoic Acid	ROS, NAD+/NADH, AMPK, OGT, PPARδ, SIRT3, TXNIP	α-Lipoic acid modulates retinal NAD-redox balance and reduces retinal cell death to ameliorate diabetic retinopathy.	Animal/Clinical	(81, 197, 198)
Melatonin	FN1, VEGF, VEGFR2, HDAC7/FOXO1/ZEB1, PI3K/AKT/Stat3/NF-κB, p38/TXNIP/NF-κB, MEG3/miR-204/Sirt1	Melatonin ameliorates diabetic retinopathy by suppressing angiogenesis and EndMT, maintaining blood-retinal barrier integrity, and relieving oxidative stress and inflammation through multiple signaling axes.	Animal/Cell	(53, 196, 199–202)
4. Terpenoids and volatile oil components				
Andrographolide (Andrographis paniculata extract)	TNF-α, IL-6, IL-1β, VEGF, Egr-1, NF-κB, IDO, GSH, ROS	Andrographolide ameliorates diabetic retinopathy via inhibiting retinal angiogenesis, inflammation and indoleamine 2,3-dioxygenase activity.	Animal/Cell	(203, 204)
Tanshinone IIA	NF-Kb, AMPK/p300, ERK, PI3K-AKT, SOD, GSH, VEGF	Tanshinone IIA alleviates diabetic retinopathy via antioxidant effects and improves retinal barrier injury by regulating the AMPK/p300/NF-κB pathway.	Animal/Cell	(205–207)
Celastrol	HIF1α/VEGF, ICAM	Celastrol suppresses high glucose-induced angiogenesis in hRECs via downregulating the HIF1α/VEGF signaling pathway.	Cell	(208)
Fructus Alpiniae zerumbet	PPAR-γ/p-CREB	EOFAZ activates PPAR-γ to suppress CaMK II-CREB signaling, downregulate GFAP and VEGF, and inhibit Müller cell gliosis against diabetic retinopathy.	Cell/Animal	(113, 209)
5. Phenylpropanoids				
Ferulic Acid	TNF-α, IL-18, IL-1β, P53, BAX, Bcl2, CAT, SOD, GSH-Px, MAPK/NF-κB	Ferulic acid protects the retina by alleviating high glucose-induced apoptosis of retinal pigment epithelial cells.	Animal/Cell	(210, 211)
6. Steroidal saponins and other saponins				
Ginsenosides (Rg1, Rb1)	HIF1α/VEGF, TNF-α, miR-2113/RP11-982M15.8/Zeb1, IRS-1/AKT/GSK3β, PI3K/AKT, AMPK-STRT1, NLRP3, SNHG7/miR-2116-5p/SIRT3, TLR4/NF-κB, ROS, miR-100-3p/FBXW7/c-MYC, ACE2/Ang1-7/Mas, VDR/cAMP/PKA/CREB	Ginsenosides exert protective effects against diabetic retinopathy by regulating multiple signaling axes, suppressing VEGF, TNF-α and NLRP3 inflammasome, inhibiting inflammation via TLR4/NF-κB pathway, restraining retinal cell proliferation, migration and angiogenesis, alleviating mesenchymal activation, fibrosis, autophagy, senescence and neuronal degeneration, improving endothelial injury through AMPK-related mechanisms, and modulating microRNA/lncRNA-mediated molecular networks as well as mitochondrial function.	Cell/Animal	(212–222)
Gypenoside	ROS, VEGF	Gypenoside inhibits retinal ferroptosis, regulates Müller cell apoptosis and autophagy, and maintains tight junction integrity.	Animal/Cell	(223–225)
7. Other characteristic plant compounds				
Gingerols (6-/8-/10-Gingerol)	NF-Kb, TNF-α, VEGF, e/iNOS, G6PDH	Gingerols ameliorate diabetic retinopathy by inhibiting inflammation, angiogenesis, apoptosis and the expression of e/iNOS and G6PDH.	Animal	(28, 226)
Allicin/S-Allyl Cysteine	PINK1/parkin, NLRP3	Allicin alleviates diabetic retinopathy by activating PINK1/Parkin-mediated mitophagy and inhibiting oxidative stress-triggered NLRP3 inflammasome.	Animal	(50)
Proanthocyanidins (Grape Seed Extract)	ROS, NLRP3, Nrf2, PI3K/AKT, Nrf2/HO-1, VEGF	Proanthocyanidins protect retinal pigment epithelial cells and alleviate diabetic retinopathy by suppressing oxidative stress and inhibiting the activation of the NLRP3 inflammasome.	Animal/Cell	(159, 227, 228)
Ganoderma lucidum Polysaccharides	Nrf2, HIF1α/VEGF	Ganoderma lucidum polysaccharides improve endothelial cell dysfunction in diabetic complications.	Animal/Cell	(229)

*Flavonoids are a subclass of polyphenols and are presented accordingly under the broader polyphenol category. Other polyphenols include non-flavonoid polyphenolic compounds. Phenylpropanoids and steroidal saponins are presented as separate categories due to their distinct chemical structures and biosynthetic pathways.

**Table 2:** Application of novel nanomaterial and advanced drug delivery systems loaded with natural antioxidants in the treatment of diabetic retinopathy.

Natural antioxidant-based drug delivery systems	Target molecule or pathway	Mechanism of action	Study type	References
Curcumin-Laden Double-Headed Nanoparticles	AGEs, PIEZO1	Curcumin-laden double-headed nanoparticles exert anti-inflammatory and anti-hyperglycemic effects, inhibit Piezo1, reduce AGEs, thereby preventing diabetic retinopathy.	Cell/Animal	(78)
Curcumin liposomes and nanocrystals	Inflammatory factor	Potential Mechanism:Curcumin liposomes and nanocrystals inhibit macrophage inflammatory polarization, reduce inflammatory factors, and attenuate inflammatory infiltration and fibrosis.	Cell/Animal	(54)
Curcumin-loaded NLCs	VEGF	Curcumin-loaded NLCs enable sustained ocular delivery, enhance corneal penetration, reduce vitreous VEGF, and preserve retinal morphology to alleviate diabetic retinopathy.	Cell/Animal	(45)
Naringenin-loaded NLCs	VEGF	Reduce vitreous VEGF, and preserve retinal morphology.	Cell/Animal	(45)
NH@NAR/β-CD	VEGF, ROS	NH@NAR/β-CD achieves sustained naringenin release, enhances solubility and antioxidant activity, targets retinal endothelial cells, and improves uptake to treat early diabetic retinopathy.	Cell	(230)
Fe-DMY NCPs	Poldip2-Nox4-H2O2, VCAM-1, HIF-1α, VEGF	Fe-DMY NCPs scavenge ROS, inhibit the Poldip2-Nox4-H_2_O_2_ pathway, downregulate VCAM-1/HIF-1α/VEGF, and alleviate retinal vascular leakage and neovascularization in diabetic retinopathy.	Cell/Animal	(61)
LPNPs	MDA, IL-1β, NLRP3, ASC, VEGF, GSH, Nrf2	Luteolin-loaded LPNPs enhance bioavailability, activate Nrf2, inhibit NLRP3/IL-1β/VEGF, reduce oxidative stress, inflammation and angiogenesis, thereby protecting retinal structure in diabetic retinopathy.	Animal	(49)
GPA-NP	Nrf2/HO-1/GPX4	GPA-NP enhances bioavailability, activates Nrf2/HO-1/GPX4 pathway by targeting Keap1, reduces oxidative stress and inflammation, and alleviates ferroptosis to protect against diabetic retinopathy.	Animal/Cell	(224)
NPS_PHE_@EOFAZ	ROS	NPS_PHE_@EOFAZ is a ROS-responsive nanoparticle that delivers EOFAZ to Müller cells, reduces oxidative stress and inflammation, and mitigates early pathological changes in diabetic retinopathy.	Cell/Animal	(113)
RSV-NS	VEGF	RSV-NS inhibits endothelial cell proliferation and migration, showing anti-angiogenic potential for treating diabetic retinopathy.	Cell	(231)
Lignin-based hydrogel*	VEGF, ROS	Lignin-based hydrogel acts as a safe ocular delivery carrier, loads natural antioxidants, inhibits VEGF, and exerts antioxidant and anti-inflammatory effects to treat diabetic retinopathy.	Animal	(110)
Chit-DC-VB12-Scu)	VEGF, VEGFR2, VWF	Chit-DC-VB12-Scu improves scutellarin bioavailability and intestinal absorption, downregulates VEGF/VEGFR2/vWF, and suppresses retinal neovascularization to ameliorate diabetic retinopathy.	Cell/Animal	(146)
SNEDDS	SIRT-1	SNEDDS act as biocompatible ocular nanocarriers to efficiently deliver resveratrol and melatonin, enhance their solubility and stability, and facilitate SIRT-1 activation for diabetic retinopathy therapy.	Cell	(79, 108)
Fe-Quer NZs	ROS, SOD, GPX, GCLC, SOD, GSH, IL-1, IL-6, TNF-α	Fe-Quer NZs mimic multiple antioxidant enzymes to scavenge ROS, alleviate oxidative stress and inflammation, inhibit angiogenesis and microvascular leakage, and exert vascular protection against early diabetic retinopathy.	Cell/Animal	(114)
SH-CDs	ROS, IL-1, IL-6, TNF-α	SH-CDs with good biocompatibility penetrate ocular tissues, scavenge reactive species, relieve oxidative stress and inflammation, and alleviate pathological damage to treat diabetic retinopathy.	Cell/Animal	(115)
AgNPs@O. europaea	TNFα, NF-κB, IL-10, GSH, CAT, SOD	AgNPs@O. europaea lowers blood glucose, enhances antioxidant levels, suppresses inflammatory TNF-α and NF-κB, elevates IL-10, and reduces retinal vascular permeability to alleviate diabetic retinopathy.	Cell/Animal	(232)
Myricetin-loaded thermoresponsive *in situ* nanoemulgel	ROS	This myricetin-loaded nanoemulgel enhances corneal permeation and retention, exerts strong antioxidant effects to treat diabetic retinopathy.	Cell	(102)
Tetrahedral Framework Nucleic Acids-Resveratrol	HIF-1, p38 MAPK, NF-κB p65	In models of hypoxia-driven retinal neovascularization, tFNAs-RSV significantly attenuated neovascular lesion size, vascular leakage, and retinal inflammatory infiltration by inhibiting HIF-1 signaling along with the p38 MAPK and NF-κB p65 pathways.	Cell/Animal	(233)

*While lignin-based hydrogels are not strictly nanomaterials in the conventional definition, they are included as an advanced drug delivery platform with nanoscale structural features that represent an emerging approach for ocular delivery of natural antioxidants.

## 2 Classification and mechanisms of natural antioxidant products

### 2.1 Types of phytochemicals

Phytochemicals, the bioactive compounds derived from plants, encompass a diverse array of chemical classes that have demonstrated significant potential in managing DR, a major microvascular complication of diabetes mellitus. Among these, polyphenols—which include the flavonoid subclass as well as other phenolic compounds—and carotenoids are the most extensively studied groups due to their potent antioxidant, anti-inflammatory, and antiangiogenic properties. Flavonoids, the most abundant polyphenols in the human diet, are found in fruits, vegetables, and medicinal herbs, and have been shown to exert antidiabetic effects by modulating oxidative stress, inflammation, and immune responses in both *in vitro* and *in vivo* models (23). Other polyphenols, including resveratrol, curcumin, and chlorogenic acid, similarly exhibit strong antioxidant activities that protect retinal cells from hyperglycemia-induced damage. Carotenoids, another major class of dietary phytochemicals, similarly exhibit strong antioxidant activities that neutralize ROS and reduce inflammation, thereby protecting retinal cells from hyperglycemia-induced damage and neovascularization characteristic of DR (24). Beyond these, other phytochemical classes such as alkaloids, terpenoids, phenylpropanoids, and saponins have been identified in polyherbal extracts with demonstrated antioxidant and anti-inflammatory activities relevant to diabetes complications (25). The detailed classification of natural antioxidants, as well as their functional targets and molecular mechanisms in the treatment of DR, are presented in **Table 1**.

Specific medicinal plants rich in these phytochemicals have been investigated for their therapeutic potential in DR. For example, Andrographis paniculata extract containing alkaloids and flavonoids was shown to improve retinal vessel diameter and enhance antioxidant enzyme levels in diabetic rats, highlighting its hypoglycemic, antioxidant, and anti-inflammatory effects (26). Similarly, extracts from Apocynum venetum leaves, rich in polyphenols including flavonoids and organic acids, protected retinal pigment epithelial cells by modulating the polyol pathway, reducing oxidative stress, and maintaining autophagy, thus preserving cellular homeostasis under hyperglycemic conditions (27). Ginger (Zingiber officinale) contains numerous phytochemicals such as gingerol and shogaol, which have demonstrated efficacy in reducing oxidative damage, inflammation, apoptosis, and pathological angiogenesis in DR models, further supporting the multifaceted roles of phytochemicals in retinal protection (28).

**Figure 2:**
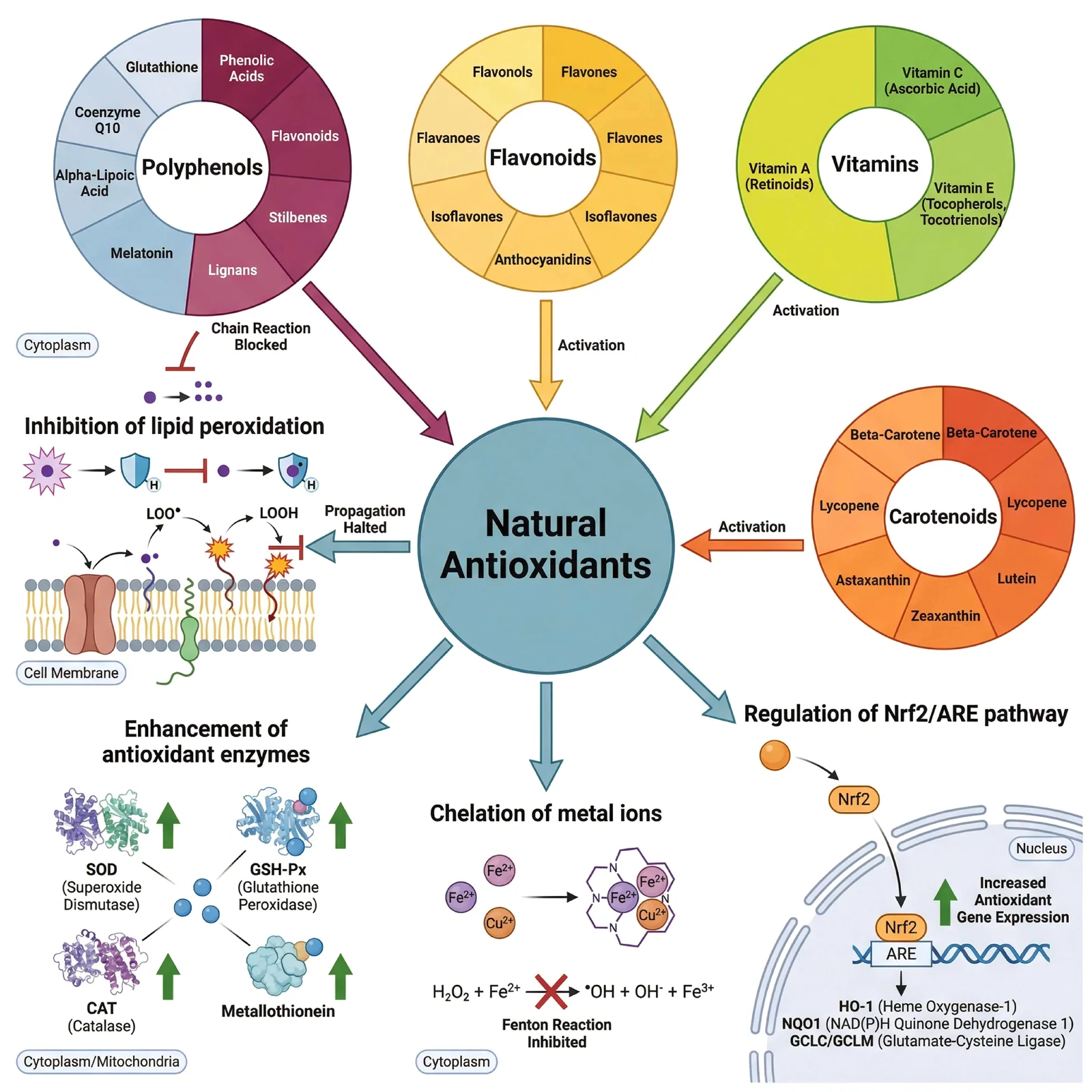
Classification and primary mechanisms of natural antioxidants. Natural antioxidants, including polyphenols, flavonoids, vitamins, and carotenoids, exert protective effects by inhibiting lipid peroxidation, enhancing antioxidant enzymes, chelating metal ions, and activating the Nrf2/ARE pathway.

Other phytochemicals with promising anti-DR activities include anthocyanins from mulberry extract, which enhance antioxidant defenses and modulate apoptosis-related proteins in retinal cells exposed to high glucose, suggesting a protective effect against oxidative injury (29). Ginkgo biloba, a traditional medicinal plant, contains flavonoids and terpenoids with neuroprotective and anti-inflammatory effects that may benefit ocular health in DR and other age-related eye diseases (30). Peperomia pellucida, rich in compounds such as dillapiole and vitexin, has shown anti-inflammatory and antiangiogenic activities via modulation of NF-κB and PPAR-γ signaling pathways in retinal cells under diabetic conditions (31, 32) (**Figure 2**).

Molecular docking and in silico studies have further identified specific phytochemicals like terchebulin, punicalagin, and chebulagic acid from medicinal plants as potential inhibitors of key molecular targets implicated in DR progression, such as the C4 protein, underscoring the therapeutic promise of these compounds (33). Moreover, polyherbal extracts combining various phytochemical classes have demonstrated synergistic antioxidant and anti-inflammatory effects, which may enhance their efficacy in managing DR (25). Despite the promising therapeutic potential of these phytochemicals, their clinical application is severely limited by poor aqueous solubility, low bioavailability, rapid metabolism, and insufficient ocular tissue penetration. Nanotechnology-based drug delivery systems have emerged as effective strategies to overcome these limitations. Encapsulation of phytochemicals in nanocarriers such as lipid nanoparticles, polymeric nanoparticles, and self-emulsifying drug delivery systems can significantly enhance their solubility, stability, and retinal targeting, thereby improving their therapeutic efficacy in DR (21, 34). These nanoformulations not only protect the active compounds from degradation but also facilitate controlled release and enhance cellular uptake in retinal tissues, addressing the critical barriers that have historically hindered the clinical translation of natural antioxidants.

### 2.2 Antioxidant mechanisms

**Figure 3:**
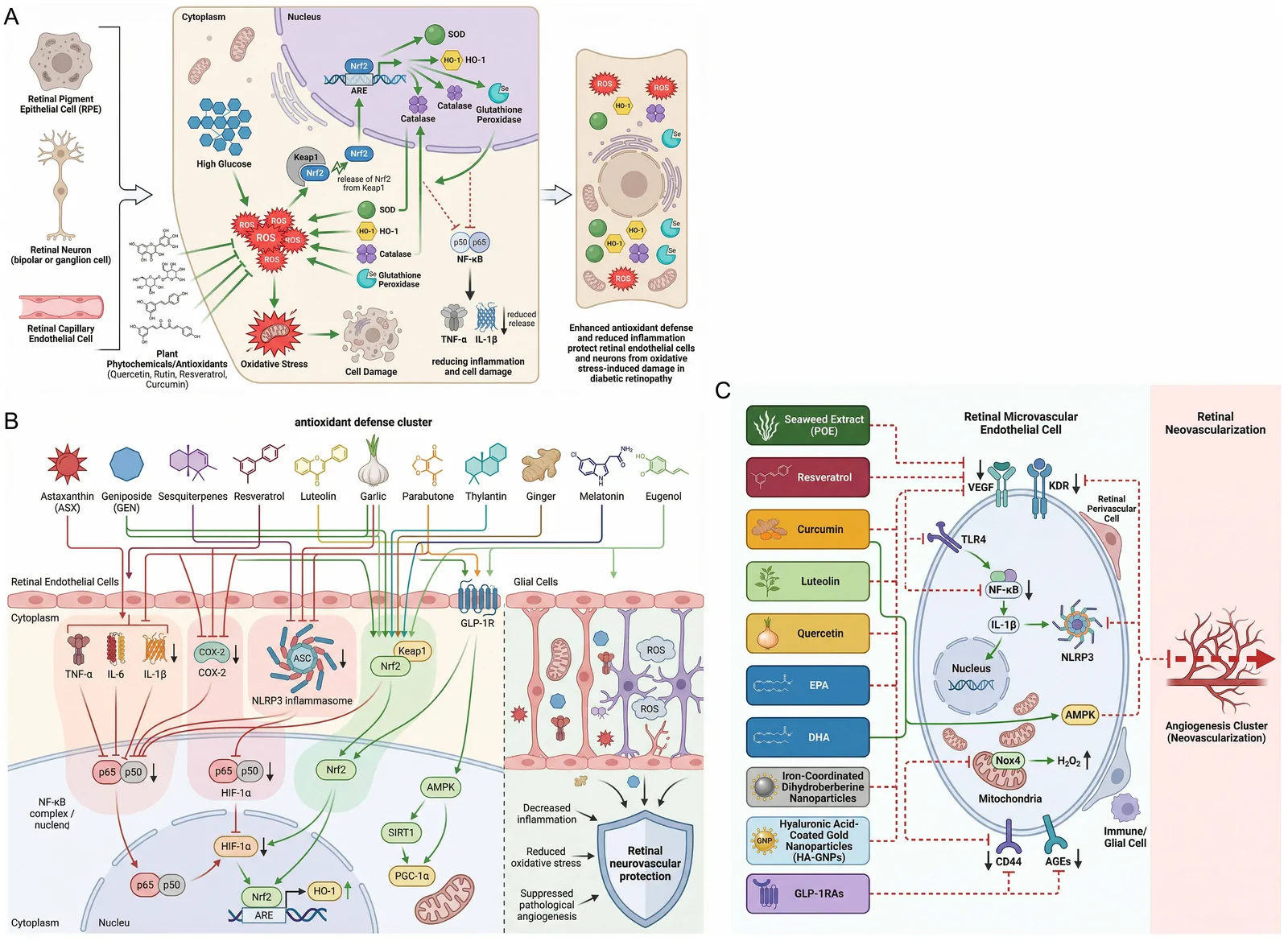
Mechanisms of phytochemical antioxidants in diabetic retinopathy. **(A)** Mechanism of the antioxidant effect. Natural antioxidants activate the Nrf2/ARE pathway to upregulate antioxidant enzymes, scavenge ROS, inhibit NF-κB-mediated inflammation, and reduce oxidative damage in endothelial, neuronal, and RPE cells. **(B)** Mechanism of the anti-inflammation effect. These compounds suppress NF-κB, NLRP3 inflammasome, and HIF-1α signaling, activate the Nrf2/HO-1 and AMPK/SIRT1 pathways, reduce oxidative stress and inflammatory cytokine release, and confer retinal neurovascular protection in endothelial and glial cells. **(C)** Mechanism of the against pathological neovascularization effect. These agents suppress VEGF/KDR, TLR4/NF-κB/NLRP3, and NOX4 signaling, activate AMPK, reduce AGEs, and inhibit retinal endothelial cell activation, thereby blocking angiogenesis.

The pathogenesis of DR is closely linked to oxidative stress, which arises from an imbalance between the production of ROS and the capacity of antioxidant defenses. Excessive ROS generation in hyperglycemic conditions leads to cellular damage in retinal tissues, contributing to vascular dysfunction, inflammation, and neuronal apoptosis. Natural antioxidants derived from plants have shown promising potential in scavenging free radicals and ROS, thereby mitigating oxidative damage in DR. For instance, alpha-lipoic acid, a naturally occurring dithiol compound, acts as a universal antioxidant by directly neutralizing ROS and regenerating other antioxidants, thus protecting retinal cells from oxidative injury. Experimental studies demonstrated that ALA prevents oxidative stress-induced retinal damage by inhibiting NF-κB activity and activating Nrf2, a master regulator of antioxidant response (15). Similarly, polyphenols such as quercetin exhibit potent free radical scavenging activity, reducing oxidative stress and inflammation in diabetic retinal tissues. Quercetin’s antioxidative effects are mediated by its ability to directly neutralize ROS and inhibit lipid peroxidation, thereby preserving retinal cell integrity (35). Moreover, procyanidins, natural polyphenolic compounds, exert strong antioxidant effects by scavenging free radicals and reducing oxidative stress markers, which are pivotal in preventing diabetic complications including retinopathy (36). Other natural antioxidants such as astaxanthin and chlorogenic acid have also been reported to scavenge ROS effectively, protecting retinal cells from oxidative damage and inflammation in diabetes (37, 38). These compounds not only neutralize ROS but also inhibit downstream pathological processes such as mitochondrial dysfunction and apoptosis, which are crucial in the progression of DR (39) (**Figure 3A**). The combined antioxidant and anti-inflammatory properties of these natural agents highlight their therapeutic potential in scavenging free radicals and ROS to prevent or delay diabetic retinal damage. However, despite encouraging preclinical evidence, clinical validation remains limited, necessitating further research to optimize their use in DR management (13, 40).

In addition to direct scavenging of ROS, natural antioxidants enhance the endogenous antioxidant defense system by upregulating the expression and activity of key antioxidant enzymes such as superoxide dismutase (SOD) and catalase (CAT). This endogenous system is critical for maintaining redox homeostasis in retinal cells under diabetic conditions. Activation of the Nrf2 pathway is a central mechanism through which many natural compounds exert this effect. Nrf2, when activated, translocates to the nucleus and binds to antioxidant response elements (ARE) in the promoter regions of genes encoding antioxidant enzymes, thereby increasing their transcription. For example, syringaresinol, a natural polyphenol, has been shown to activate Nrf2, leading to enhanced expression of antioxidant enzymes and suppression of oxidative stress in DR models. This activation also downregulates hypoxia-inducible factor-1α (HIF-1α) and VEGF, mitigating retinal microvascular damage (41). Similarly, carnosol protects human retinal endothelial cells from high glucose-induced oxidative damage by inducing heme oxygenase-1 (HO-1) expression via Nrf2 activation, which involves the ERK1/2 signaling pathway, thereby enhancing cellular antioxidant capacity (18). Pterostilbene, another natural antioxidant, improves redox status and restores the activities of antioxidant enzymes in diabetic retinal tissue through activation of the PI3K/AKT/GSK3β/Nrf2 pathway, demonstrating a potent protective effect against early DR alterations (16). Curcumin, a well-studied polyphenol, also modulates the Nrf2 pathway, maintaining its homeostasis and promoting antioxidant enzyme expression, which alleviates oxidative stress and retinal injury in diabetic models (42). Beyond Nrf2, other signaling pathways such as AMP-activated protein kinase (AMPK) and sirtuin 1 (SIRT1) are implicated in the upregulation of antioxidant enzymes by natural compounds like resveratrol, further contributing to oxidative stress reduction and improved retinal health in diabetes (15, 43). The enhancement of endogenous antioxidant defenses not only reduces ROS levels but also attenuates inflammation and apoptosis, key contributors to DR progression. While these molecular insights underscore the therapeutic promise of natural antioxidants in boosting intrinsic antioxidant enzymes, challenges such as bioavailability and targeted delivery remain. Nonetheless, ongoing advances in nutraceutical formulations and nanotechnology hold potential to overcome these barriers and optimize the clinical application of natural antioxidants in DR (12, 44).

The enhancement of endogenous antioxidant defenses by natural compounds is significantly amplified when these agents are delivered using nanotechnology-based systems. Nanocarriers can protect labile antioxidants from degradation, facilitate their intracellular delivery, and enable sustained release, thereby potentiating their effects on the Nrf2 pathway and antioxidant enzyme expression. For example, curcumin-loaded nanostructured lipid carriers (NLCs) have demonstrated superior Nrf2 activation and HO-1 upregulation compared to free curcumin in retinal endothelial cells, attributed to enhanced cellular uptake and sustained intracellular drug levels (45). These nanoformulations not only improve the bioavailability of natural antioxidants but also enable their targeted delivery to retinal tissues, maximizing therapeutic efficacy while minimizing systemic side effects. The integration of nanocarrier technology with natural antioxidant therapy thus represents a promising strategy to overcome the pharmacokinetic barriers that have limited the clinical translation of these compounds.

### 2.3 Anti-inflammatory effects

Inflammation plays a central role in the pathogenesis and progression of DR, a major microvascular complication of diabetes mellitus. Chronic hyperglycemia induces oxidative stress and triggers the release of pro-inflammatory cytokines such as tumor necrosis factor-alpha (TNF-α) and interleukin-6 (IL-6), which exacerbate retinal damage by promoting leukostasis, vascular permeability, and neuronal apoptosis. Natural antioxidant compounds have demonstrated significant anti-inflammatory effects by downregulating these key inflammatory mediators, thereby mitigating retinal inflammation and preserving retinal structure and function. For example, studies have shown that astaxanthin (ASX), a potent natural antioxidant, reduces retinal levels of TNF-α, IL-1β, and IL-6 in streptozotocin-induced diabetic rat models, concomitant with decreased oxidative stress and apoptosis in retinal tissues. This effect is mediated through activation of the Nrf2/Keap1 antioxidant pathway, which suppresses inflammation and oxidative damage (46). Similarly, geniposide (GEN), a natural anti-inflammatory compound, inhibits NF-κB activation and reduces secretion of inflammatory cytokines in retinal Müller cells exposed to high glucose, with effects dependent on Nrf2 signaling and glucagon-like peptide-1 receptor (GLP-1R) activation (47). Another example is sesamin, a natural lignan, which dose-dependently suppresses TNF-α, IL-1β, and IL-6 expression in retinal microglia under hyperglycemic conditions, thereby alleviating microglial inflammation and blood-retinal barrier breakdown in diabetic mice (44). Resveratrol, a polyphenol with well-documented anti-inflammatory properties, inhibits the expression of TNF-α, IL-6, and cyclooxygenase-2 (COX-2) in diabetic retinas, improving retinal thickness and visual function through modulation of SIRT1/NF-κB and Nrf2 pathways (48). Luteolin, a flavonoid, also demonstrates anti-inflammatory effects by reducing retinal levels of TNF-α and IL-6, although its clinical application is limited by poor solubility; novel nanocarrier formulations have been developed to enhance its bioavailability and therapeutic efficacy in DR models (49). Moreover, natural compounds such as allicin from garlic exhibit anti-inflammatory actions by downregulating inflammasome components (e.g., NLRP3) and pro-inflammatory cytokines, partly through activation of mitophagy pathways, which attenuates pyroptosis and oxidative stress in diabetic retinas (50). Phytochemicals like palbinone and tilianin have been reported to inhibit NLRP3 inflammasome activation and reduce inflammatory cytokines in diabetic retinas, contributing to antioxidant and anti-inflammatory protection (51, 52). Additionally, natural extracts such as ginger have been shown to suppress ocular expression of NF-κB and VEGF, decrease TNF-α levels, and improve oxidative stress markers, collectively reducing inflammation and angiogenesis in DR (28). The anti-inflammatory effects of these natural antioxidants often involve modulation of key signaling pathways, including Nrf2/HO-1, AMPK/Sirt1/PGC-1α, and NF-κB, which regulate the balance between pro- and anti-inflammatory mediators. For instance, melatonin attenuates retinal inflammation by activating the Sirt1 pathway, leading to deacetylation and suppression of NF-κB activity in Müller cells (53). Syringaresinol alleviates early DR by activating Nrf2 and downregulating hypoxia-inducible factor-1α (HIF-1α)/VEGF, thereby reducing inflammation and oxidative stress in retinal microvasculature (41) (**Figure 3B**). Collectively, these findings underscore the therapeutic potential of natural antioxidant compounds in mitigating retinal inflammation in DR by inhibiting the expression of inflammatory cytokines such as TNF-α and IL-6. This anti-inflammatory action complements their antioxidant capacity, ultimately protecting retinal cells from hyperglycemia-induced damage and preserving visual function. Further clinical studies and optimization of delivery systems are warranted to translate these promising preclinical results into effective treatments for DR.

The anti-inflammatory efficacy of natural antioxidants is critically dependent on their ability to reach sufficient concentrations at the target retinal tissues, which is often hindered by poor bioavailability. Advanced nanocarrier systems address this limitation by enhancing the ocular penetration and sustained release of anti-inflammatory compounds. For instance, luteolin-loaded lipid-polymer hybrid nanoparticles (LPNPs) functionalized with TPGS have shown significantly enhanced oral bioavailability and retinal tissue distribution compared to free luteolin, leading to superior inhibition of NLRP3 inflammasome activation and inflammatory cytokine production in diabetic models (49). The nanoformulation achieved prolonged drug release, improved intestinal permeability, and substantial attenuation of retinal inflammatory markers, demonstrating the translational potential of nanocarrier systems. Similarly, geniposide-loaded nanoparticles and curcumin SNEDDS formulations have demonstrated enhanced intracellular uptake in retinal cells, resulting in more effective suppression of NF-κB activation and inflammatory mediator expression (54). These examples underscore the critical role of nanotechnology in unlocking the full anti-inflammatory potential of natural antioxidants for DR therapy.

### 2.4 Anti-angiogenesis

Pathological neovascularization is a hallmark of DR, driven primarily by the overexpression of VEGF, which promotes aberrant angiogenesis and vascular leakage in the retina. Natural antioxidants have emerged as promising agents to inhibit this pathological process by targeting VEGF and its downstream signaling pathways. For instance, Posidonia oceanica extract (POE), derived from a Mediterranean seagrass, has demonstrated potent anti-angiogenic effects by selectively disrupting VEGF-induced signaling in human endothelial colony-forming cells (ECFCs). POE inhibits phosphorylation of VEGF receptor 2 (KDR), mammalian target of rapamycin (mTOR), and extracellular signal-regulated kinase (ERK), thereby impairing endothelial cell migration, invasion, and tube formation essential for neovascularization, without compromising cell viability. Additionally, POE reduces intracellular ROS accumulation and downregulates redox-regulating genes, linking its antioxidant capacity to anti-angiogenic activity (55). Similarly, resveratrol, a natural polyphenol, exhibits multi-modal protective effects in DR, including suppression of pathological neovascularization. It modulates oxidative stress and inflammatory pathways, ultimately inhibiting VEGF expression and angiogenic signaling. However, its clinical translation is challenged by poor ocular bioavailability, prompting exploration of advanced drug delivery systems to enhance retinal targeting (56). Curcumin, another natural compound with antioxidant and anti-inflammatory properties, protects retinal pigment epithelium (RPE) cells under hyperglycemic conditions by preserving tight junction integrity and inhibiting retinal neovascularization. It achieves this by modulating key signaling pathways such as NF-κB and TLR4, which are involved in inflammation and angiogenesis, thus preventing VEGF-mediated vascular proliferation (57, 58). Luteolin-loaded nanocarriers have also shown promise in reducing retinal VEGF expression and angiogenesis in diabetic models. By enhancing luteolin’s bioavailability, these nanoplatforms effectively decrease oxidative stress and inflammatory markers, including IL-1β and NLRP3 inflammasome components, while downregulating VEGF, thereby mitigating neovascular complications (49). Quercetin, a flavonoid abundant in fruits and vegetables, inhibits retinal neovascularization by reducing oxidative stress and inflammatory cytokines, and by modulating signaling pathways such as AMP-activated protein kinase (AMPK), which influence VEGF expression. Despite its therapeutic potential, quercetin’s clinical use is limited by low bioavailability, necessitating further formulation optimization (59). Omega-3 fatty acids, including eicosapentaenoic acid (EPA) and docosahexaenoic acid (DHA), contribute to retinal health by exerting anti-angiogenic effects that inhibit abnormal blood vessel growth characteristic of proliferative DR. Their antioxidative and anti-inflammatory mechanisms reduce VEGF-driven neovascularization, highlighting their preventive role in retinal diseases (60) (**Figure 3C**). Furthermore, innovative nanotechnology approaches, such as iron-coordinated dihydromyricetin nanoparticles, have been developed to target oxidative stress and angiogenesis simultaneously. These nanoparticles downregulate the Poldip2-Nox4-H2O2 pathway and reduce expression of VEGF and other angiogenic factors, effectively alleviating retinal vascular leakage and neovascularization in diabetic models (61). Gold nanoparticles coated with hyaluronic acid (HA-GNPs) represent another advanced strategy for ocular drug delivery. HA enhances nanoparticle stability and retinal targeting via CD44 receptor interaction, enabling effective inhibition of AGEs-mediated retinal pigment epithelial cell death and neovascularization. This approach underscores the potential of combining natural compounds with nanocarriers to improve anti-angiogenic therapy in DR (62). These nanocarrier-based approaches offer significant advantages over conventional formulations, including enhanced stability, targeted delivery, reduced systemic toxicity, and the potential for combination therapy with multiple natural compounds. Lastly, glucagon-like peptide-1 receptor agonists (GLP-1RAs), though not classical antioxidants, exhibit anti-angiogenic properties by stabilizing vascular integrity and modulating extracellular matrix homeostasis, contributing to reduced neovascular complications in DR (63). Collectively, these findings highlight that natural antioxidants and their advanced formulations can effectively inhibit VEGF expression and signaling pathways, thereby preventing pathological neovascularization in DR. This multi-targeted approach addresses oxidative stress, inflammation, and angiogenesis, offering promising therapeutic avenues for early intervention and management of DR.

## 3 Research progress of major natural antioxidant products in DR

### 3.1 Quercetin

**Figure 4:**
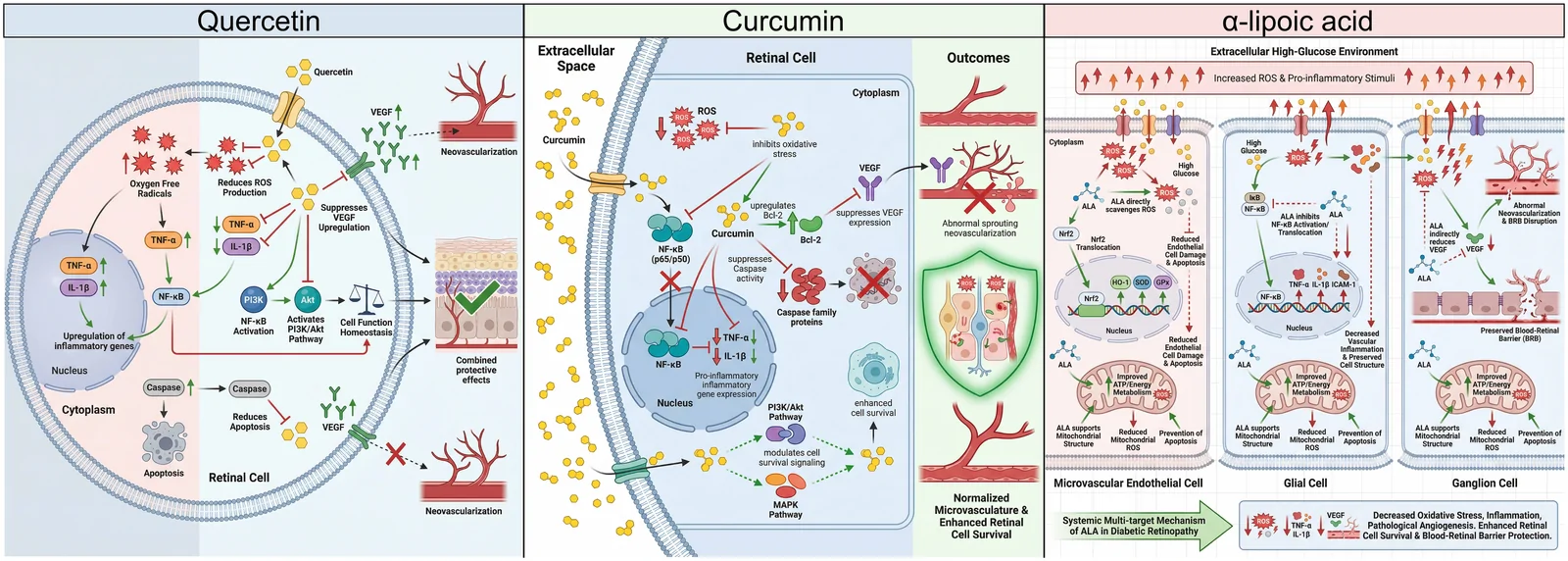
Comparative schematic of the molecular mechanisms of quercetin, curcumin, and α-lipoic acid (ALA) in diabetic retinopathy. All three natural antioxidants exert multi-target protective effects: they scavenge ROS, inhibit NF-κB-mediated inflammation and VEGF-driven neovascularization, and regulate cell survival/apoptosis pathways. Quercetin additionally activates PI3K/AKT signaling; curcumin modulates PI3K/AKT/MAPK pathways and upregulates Bcl-2; ALA further activates the Nrf2 antioxidant pathway, preserves mitochondrial function, and protects the blood-retinal barrier across endothelial, glial, and ganglion cells.

Quercetin, a naturally occurring flavonoid abundant in fruits and vegetables, has garnered extensive attention for its multifaceted therapeutic potential in DR, a leading cause of vision impairment in diabetic patients. Experimental studies across various animal models consistently demonstrate quercetin’s capacity to mitigate retinal injury and preserve visual function. For instance, in a zebrafish model of DR induced by streptozotocin (STZ), nano-formulated quercetin (NQ) administered intraperitoneally for 21 days attenuated retinal damage markers, including normalization of eyeball-to-body weight ratio and improvement in behavioral visual responses such as optomotor and phototactic responses. Biochemical assays revealed that NQ reduced plasma glucose and homocysteine levels, decreased retinal lipid peroxidation, and restored antioxidant enzyme activities, effects comparable to dexamethasone treatment, underscoring its antioxidative and anti-hyperglycemic properties (64). In rodent models, quercetin administration increased retinal cell layer thickness and ganglion cell counts, while suppressing pro-inflammatory cytokines (IL-1β, IL-6, TNF-α), high mobility group box-1, and NLRP3 inflammasome activation. This anti-inflammatory effect was linked to upregulation of heme oxygenase-1, a cytoprotective enzyme, as inhibition of this pathway abrogated quercetin’s protective effects (65). At the cellular level, quercetin inhibited angiogenesis in human retinal microvascular endothelial cells exposed to high glucose by downregulating the NLRP3 inflammasome components (NLRP3, ASC, Caspase-1) and pro-inflammatory interleukins (IL-1β, IL-18), while modulating autophagy-related proteins LC3 and Beclin-1, suggesting a role in controlling pathological neovascularization and inflammation (66). Network pharmacology analyses of traditional Chinese medicine formulations containing quercetin identified key targets such as IL6, EGFR, CASP3, and VEGFA, implicating quercetin in regulating AGE-RAGE, TNF, HIF-1, and VEGF signaling pathways central to DR pathogenesis (67). Mechanistically, quercetin exerts antioxidant effects by scavenging ROS, inhibiting myeloperoxidase-dependent hypochlorous acid generation, and enhancing endogenous antioxidant defenses including glutathione and superoxide dismutase, thereby protecting retinal endothelial cells from oxidative damage (39, 68). Furthermore, quercetin modulates cellular senescence and inflammation by targeting the NLRP3 inflammasome and related signaling pathways such as NF-κB, AMPK, and SIRT1, which are implicated in DR progression (69, 70) (**Figure 4**). Combinational therapies pairing quercetin with conventional antidiabetic drugs like gliclazide have demonstrated synergistic effects in glycemic control and pancreatic β-cell protection, further supporting its adjunctive use (71). Collectively, these studies underscore quercetin’s significant capacity to reduce retinal damage, inhibit inflammation and neovascularization, and improve visual outcomes in DR models, positioning it as a promising natural antioxidant agent for DR management pending further clinical validation.

Despite its promising preclinical efficacy, clinical translation of quercetin faces challenges due to poor bioavailability, low aqueous solubility, and rapid metabolism. Advances in nanoformulation techniques have been instrumental in overcoming these limitations. Quercetin nanoemulsions, phytosome complexes, and lipid-based nanocarriers have been developed to improve solubility, stability, and targeted delivery to retinal tissues (64, 72). Nano-formulated quercetin (NQ) has demonstrated superior retinal protection in zebrafish and rodent models compared to free quercetin, with enhanced antioxidant enzyme activity and improved behavioral visual responses (64). Quercetin-loaded thermoresponsive *in situ* nanoemulgels have also been explored to enhance corneal permeation and retention for topical ocular administration (73). These nanoformulations not only improve the pharmacokinetic profile of quercetin but also enable sustained release and targeted accumulation in retinal tissues, representing a promising strategy to translate the therapeutic potential of this flavonoid into clinical practice for DR management.

### 3.2 Curcumin

Curcumin, a natural polyphenol derived from the rhizome of Curcuma longa, has garnered significant attention for its multifaceted pharmacological properties, including potent antioxidant, anti-inflammatory, and anti-apoptotic effects, which are highly relevant in the context of DR. DR is a microvascular complication of diabetes mellitus characterized by oxidative stress, inflammation, and retinal neurovascular damage, ultimately leading to vision impairment and blindness. The Nrf2/HO-1 signaling pathway plays a pivotal role in cellular defense against oxidative stress by regulating the expression of antioxidant enzymes and cytoprotective proteins. Curcumin’s therapeutic potential in DR has been increasingly attributed to its ability to activate this pathway, thereby mitigating oxidative damage and inflammation in retinal tissues.

Several preclinical studies have demonstrated that curcumin administration in diabetic models significantly alleviates retinal oxidative stress by enhancing Nrf2 nuclear translocation and upregulating heme oxygenase-1 (HO-1) expression. For instance, in streptozotocin-induced diabetic rats, curcumin treatment reduced retinal edema and photoreceptor apoptosis more effectively than insulin alone, correlating with a restoration of Nrf2 pathway homeostasis. This suggests that curcumin’s antioxidant capacity not only scavenges ROS but also modulates endogenous antioxidant defenses through Nrf2/HO-1 activation, resulting in decreased oxidative stress and improved retinal structural integrity (42) (**Figure 4**). Moreover, transcriptomic analyses revealed that curcumin suppresses the AGE-RAGE signaling pathway and extracellular matrix-receptor interactions, both critical in DR pathogenesis, further supporting its multifactorial protective effects.

Clinical investigations have also begun to explore curcumin’s efficacy in DR patients. A randomized, double-blind controlled trial administering curcumin-piperine supplementation reported significant improvements in total antioxidant capacity and superoxide dismutase levels, alongside reductions in malondialdehyde and creatinine, markers indicative of oxidative stress and renal function, respectively. Although no significant changes were observed in inflammatory markers or retinal vascular density within the study period, these findings underscore curcumin’s potential to enhance systemic antioxidant defenses in non-proliferative DR (74). Additionally, curcumin’s anti-inflammatory effects have been documented in human vitreous samples from DR patients, where combined treatment with curcumin, homotaurine, and vitamin D3 significantly reduced pro-inflammatory cytokines such as TNF-α and IL-2, suggesting synergistic modulation of inflammatory mediators involved in DR progression (75).

At the molecular level, curcumin’s activation of the Nrf2/HO-1 axis involves the promotion of Nrf2 nuclear translocation, which binds to antioxidant response elements (ARE) in the promoter regions of genes encoding antioxidant enzymes, including HO-1. HO-1, in turn, exerts cytoprotective effects by degrading pro-oxidant heme into biliverdin, carbon monoxide, and free iron, thereby reducing oxidative damage. Curcumin also modulates upstream signaling pathways such as PI3K/AKT and MAPK, which influence Nrf2 activation, and suppresses NF-κB-mediated inflammatory responses, creating a comprehensive antioxidative and anti-inflammatory milieu in retinal cells (57, 76) (**Figure 4**). Furthermore, curcumin’s neuroprotective effects in the retina are linked to the preservation of tight junction integrity in retinal pigment epithelial cells and inhibition of retinal neovascularization, both critical factors in DR pathophysiology (57).

Despite its promising biological activities, curcumin’s clinical application is limited by poor aqueous solubility, low bioavailability, rapid metabolism, and systemic elimination. To overcome these challenges, various delivery systems such as nanoemulsions, nanoparticles, and cyclodextrin inclusion complexes have been developed, enhancing curcumin’s stability, ocular penetration, and sustained release. For example, curcumin-loaded NLCs and SNEDDS have demonstrated improved retinal bioavailability and antioxidant efficacy in diabetic models, supporting their potential for clinical translation in DR management (45, 77). Additionally, combination therapies incorporating curcumin with insulin or other natural antioxidants have shown synergistic effects in maintaining Nrf2 pathway homeostasis and reducing retinal injury (42, 78).

In summary, curcumin exerts protective effects against DR primarily through the modulation of the Nrf2/HO-1 signaling pathway, leading to enhanced antioxidant defenses, reduced oxidative stress, and attenuation of inflammatory responses in retinal tissues. Its multifaceted actions extend to preserving retinal structure and function, inhibiting pathological angiogenesis, and improving metabolic parameters associated with diabetes. Although clinical evidence remains limited, advancements in curcumin delivery technologies and combination therapies hold promise for its effective incorporation into DR treatment regimens. Future large-scale, well-designed clinical trials are warranted to validate curcumin’s efficacy and safety as a complementary therapeutic agent targeting the oxidative and inflammatory mechanisms underlying DR.

Despite its promising biological activities, curcumin’s clinical application is severely limited by poor aqueous solubility, low bioavailability, rapid metabolism, and systemic elimination. To overcome these challenges, a wide range of delivery systems has been developed, including nanoemulsions, nanoparticles, liposomes, NLCs, and cyclodextrin inclusion complexes. Curcumin-loaded NLCs have demonstrated enhanced retinal bioavailability and antioxidant efficacy in diabetic models, with improved corneal penetration, reduced vitreous VEGF levels, and preserved retinal morphology (45). Similarly, curcumin SNEDDS have shown superior physicochemical stability, mucoadhesive properties, and enhanced intracellular uptake in retinal pigment epithelial cells, resulting in more potent antioxidant activity and induction of protective SIRT1 expression compared to free curcumin (79). Curcumin-laden double-headed nanoparticles have been developed to combine anti-inflammatory and anti-hyperglycemic effects, inhibiting Piezo1 and reducing AGEs in rodent models (78). These advanced delivery platforms represent a significant advancement in overcoming the translational barriers associated with curcumin, supporting its potential for clinical application in DR management.

### 3.3 α-Lipoic acid

α-Lipoic acid (ALA) is a naturally occurring dithiol compound that functions as a vital cofactor for mitochondrial enzyme complexes, playing a central role in cellular energy metabolism. Due to its unique chemical structure, ALA exhibits potent antioxidant properties and is often referred to as a “universal antioxidant” because it can directly scavenge ROS and regenerate other antioxidants such as vitamins C and E, coenzyme Q10, and glutathione. This broad antioxidant capacity makes ALA particularly attractive for addressing oxidative stress, a key pathological mechanism underlying diabetic complications including DR (15, 80). Oxidative stress in diabetes arises primarily from chronic hyperglycemia-induced overproduction of ROS, activation of the polyol pathway, and formation of AGEs, all of which contribute to retinal microvascular damage, inflammation, and neuronal dysfunction (**Figure 4**).

Experimental studies have demonstrated that ALA can mitigate oxidative damage in retinal tissues by inhibiting pro-inflammatory signaling pathways such as NF-κB and by activating protective transcription factors like Nrf2 and AMP-activated protein kinase (AMPK) in retinal ganglion cells. These molecular effects translate into reduced retinal oxidative stress and inflammation, thereby preserving retinal structure and function (15) (**Figure 4**). Clinically, supplementation with ALA, often in combination with other antioxidants such as genistein and vitamins, has shown promise in pre-retinopathic diabetic patients by protecting retinal cells and attenuating inflammatory responses, suggesting a potential role in early intervention for DR (15).

Moreover, clinical trials have reported improvements in visual function parameters, including oscillatory potentials and contrast sensitivity, following ALA supplementation in diabetic patients, highlighting its beneficial effects on retinal electrophysiology and visual performance (80). A noteworthy aspect is the chronopharmacological optimization of ALA administration; studies in diabetic rat models demonstrated that timing the administration of ALA alongside other antidiabetic drugs enhanced glycemic control and retinal antioxidant profiles, resulting in histological preservation of retinal tissue comparable to non-diabetic controls (81). This suggests that ALA’s efficacy may be maximized by aligning its dosing with circadian rhythms, an important consideration for clinical translation. Despite its therapeutic potential, ALA’s clinical application is somewhat limited by its low water solubility and bioavailability. Innovative drug delivery systems, such as erodible polymeric inserts based on Eudragit films, have been developed to enhance transcorneal permeability and retention of ALA in ocular tissues, thereby improving its local bioavailability and therapeutic effect for ocular complications in diabetes (82). This advancement could facilitate more effective topical or localized delivery of ALA for DR and other ocular surface diseases.

In addition to its antioxidant effects, ALA exhibits anti-inflammatory and metal-chelating properties, which contribute to its neuroprotective effects in diabetic neuropathies and ocular surface diseases such as dry eye disease, conditions commonly comorbid with diabetes (83). By reducing oxidative damage and inflammation in the meibomian glands and corneal epithelium, ALA may improve tear film stability and ocular surface health, indirectly supporting retinal function in diabetic patients. Current clinical evidence supports its potential to improve retinal function and prevent progression of diabetic ocular complications, further large-scale, long-term randomized controlled trials are necessary to establish optimal dosing regimens, delivery methods, and confirm its efficacy and safety profile in diverse diabetic populations. Integrating ALA supplementation with conventional diabetes management may offer a promising strategy to reduce the burden of DR and preserve vision in affected patients (40, 84).

Despite its therapeutic potential, ALA’s clinical application is limited by its low water solubility, rapid clearance, and poor bioavailability, which restrict its effective retinal concentrations. Innovative drug delivery systems have been developed to address these limitations. Erodible polymeric inserts based on Eudragit films have been designed to enhance transcorneal permeability and retention of ALA in ocular tissues, thereby improving its local bioavailability and therapeutic effect for ocular complications in diabetes (82). Polymeric nanoparticles and liposomal formulations of ALA have also been explored to protect the compound from degradation, enable sustained release, and facilitate targeted delivery to retinal tissues (85). These advanced delivery systems can significantly enhance the ocular bioavailability of ALA, making it a more effective therapeutic option for DR and other diabetic complications. The development of sustained-release formulations is particularly important for ALA, as its rapid clearance necessitates frequent administration for maintaining therapeutic concentrations. Nanotechnology-based approaches offer the potential to overcome this limitation and optimize the clinical use of ALA in DR management.

## 4 Clinical applications and challenges

### 4.1 Clinical trial results

Clinical trials investigating the efficacy of natural antioxidants in DR have increasingly highlighted their potential to improve retinal blood flow and retinal function, addressing key pathological mechanisms underlying DR. Alpha-lipoic acid, a naturally occurring dithiol compound with potent antioxidant properties, has been evaluated in clinical settings involving pre-retinopathic diabetic patients. Trials combining ALA with genistein and vitamins demonstrated protective effects on retinal cells and a reduction in inflammatory responses, suggesting that ALA can mitigate oxidative stress and inflammation in the diabetic retina, thereby preserving retinal function (15). Similarly, dietary anthocyanins, a subgroup of polyphenols abundant in fruits and vegetables, have been clinically shown to improve glucose control, reduce oxidative stress, and enhance insulin sensitivity in diabetic patients, which may indirectly benefit retinal microvascular health and function (86). Despite the promising biochemical and physiological effects observed, clinical studies remain limited in number and often vary in design, dosage, and duration, which affects the consistency of outcomes.

Carotenoids such as lutein and zeaxanthin, known for their antioxidant and anti-inflammatory properties, have been assessed in clinical trials for their role in maintaining retinal integrity and function. These compounds are concentrated in the macula and are believed to protect the retina from oxidative damage induced by hyperglycemia and light exposure. Clinical evidence suggests that supplementation with macular xanthophylls can improve retinal health and delay the progression of DR, although larger-scale randomized controlled trials are needed to confirm these benefits (87, 88). Furthermore, clinical trials with flavonoids and polyphenols such as quercetin and resveratrol have indicated improvements in oxidative stress markers and retinal function parameters, supporting their therapeutic potential in DR management (35, 43).

Nutraceuticals, defined as natural functional foods with minimal side effects, have also been explored in clinical contexts for DR. While preclinical studies show their antioxidative, anti-inflammatory, neuroprotective, and vasoprotective effects, clinical trials have yielded mixed results, largely due to issues such as low bioavailability and instability of these compounds. Innovative delivery systems, including nanoparticle carriers, are being developed to overcome these limitations and enhance clinical efficacy (89, 90). Notably, fenofibrate, a lipid-lowering agent with antioxidant and anti-inflammatory properties, has shown in clinical trials to slow DR progression independently of its lipid-modifying effects, highlighting the importance of antioxidant mechanisms in retinal protection (91, 92).

Epidemiological studies further support the association between higher antioxidant exposure and reduced risk and severity of retinopathy. A higher oxidative balance score, reflecting cumulative antioxidant intake and lifestyle factors, correlates with a lower prevalence of retinopathy and decreased all-cause mortality among affected individuals, underscoring the clinical relevance of antioxidant status in DR outcomes (93). Additionally, clinical investigations into trace elements such as zinc have revealed that supplementation may improve glycemic control and reduce the incidence of diabetic complications including retinopathy, suggesting a supportive role for micronutrient antioxidants in clinical management (94).

**Table 3** summarizes the key clinical studies of natural antioxidants in DR. Quercetin combined with resveratrol reduced central macular thickness by 32 μm, and improved visual acuity by 0.15 logMAR (95, 96). NHANES analysis associated flavonoid-rich diets with a 30% DR risk reduction and improved glycemic and inflammatory markers (97). Curcumin-piperine supplementation enhanced total antioxidant capacity and SOD, reduced MDA, and improved mental health and sleep quality, though inflammatory and retinal vascular changes were not significant (74, 98); complementary *in vitro* evidence showed curcumin combination reduced TNF-α and IL-2 in human vitreous (75). Lutein/zeaxanthin supplementation improved contrast sensitivity (p=0.02) in a 31-patient RCT (99), while a larger trial (n=208) reported DR progression in 61% of supplemented patients vs. 91% of controls, with reduced lipid peroxidation (100). α-Lipoic acid (600 mg/day, 24 months) stabilized DR progression, reduced macular edema, and maintained visual function (101). Collectively, these studies provide encouraging yet preliminary evidence.

**Table 3:** Clinical studies of natural antioxidants in diabetic retinopathy.

Natural antioxidant	Study design	Population	Intervention	Duration	Key outcomes	References
Quercetin + Resveratrol	RCT (part of combination)	DR patients (n=85)	Quercetin 500 mg/day + resveratrol 250 mg/day	6 months	↓ Central macular thickness by 32 μm; ↓ HbA1c by 0.7%; ↑ Visual acuity by 0.15 logMAR	(96)
Flavonoid-rich fruit and vegetable	Analysis of NHANES 2003–2006	n=381 diabetic patient	N/A	N/A	↓ risk of developing DR by 30%;↓ C-reactive protein levels, HbA1C and glucose	(97)
Curcumin + Piperine	RCT, double-blind, placebo-controlled	Non-proliferative DR patients (n=60)	Curcumin 1000 mg + piperine 10 mg/day	12 weeks	↑ Total antioxidant capacity; ↑ SOD; ↓ MDA; ↑ Mental health; ↑ Sleep quality	(74, 98)
Curcumin + Homotaurine + Vitamin D3	*In vitro* (human vitreous)	DR patients (n=15)	Combined treatment	N/A	↓ TNF-α; ↓ IL-2	(75)
Lutein/Zeaxanthin	RCT, double-blind, placebo-controlled	Non-proliferative DR patients (n=31)	Lutein 10 mg/day	36 weeks	↑ Contrast sensitivity at 3 cycles/degree (p=0.02); Slight improvement in VA; Glare sensitivity: NS	(99)
Lutein/Zeaxanthin (combination in supplements)	RCT, prospective, multicenter	T2DM with NPDR ± DME (n=208)	Nutrof Omega^®^ (lutein, zeaxanthin, omega-3)	18 months	DR progression: 61% (supplemented) vs. 91% (control); ↓ Plasma lipid peroxidation	(100)
α-Lipoic Acid (ALA)	Clinical trial	T2DM with mild-moderate NPDR (n=47)	ALA 600 mg/day	24 months	Stabilization of DR progression; ↓ New macular oedema cases; Stable vision and contrast sensation	(101)

The symbols ↓ and ↑ indicate a decrease or increase, respectively, in the corresponding parameter in the treatment group of the experiment.

Despite these encouraging findings, the clinical translation of natural antioxidants in DR is challenged by heterogeneity in study designs, small sample sizes, and limited long-term data. Many trials emphasize surrogate endpoints such as oxidative stress biomarkers or retinal imaging parameters rather than hard clinical outcomes like visual acuity or progression to proliferative DR. Therefore, well-designed, large-scale randomized controlled trials with standardized antioxidant formulations and optimized delivery methods are essential to validate efficacy, determine optimal dosing, and establish safety profiles. In conclusion, current clinical trial evidence supports the beneficial role of natural antioxidant products in improving retinal blood flow and function in DR patients, but further rigorous clinical research is needed to fully integrate these agents into standard care protocols. Multi-targeted strategies, potentially enhanced by nanotechnology-based delivery, warrant future investigation.

### 4.2 Challenges in application

Despite the promising antioxidant, anti-inflammatory, and antiangiogenic properties of natural compounds in DR management, significant challenges hinder their clinical translation, particularly low bioavailability and dose standardization. Many phytochemicals such as curcumin, resveratrol, quercetin, and ALA exhibit poor aqueous solubility, rapid metabolism, and limited stability, resulting in suboptimal systemic and ocular tissue concentrations after oral or topical administration. For instance, curcumin is known for its potent antioxidative and anti-inflammatory effects but suffers from poor bioavailability, necessitating the use of adjuvants like piperine or advanced delivery systems such as nanoemulsions to enhance absorption and retinal tissue penetration (14, 102). Similarly, resveratrol’s therapeutic potential is limited by its rapid metabolism and low systemic availability, prompting research into nanoparticle-based delivery to improve its ocular bioavailability (22, 103). Alpha-lipoic acid, despite its “universal antioxidant” status and demonstrated benefits in diabetic ocular complications, faces challenges due to low water solubility and rapid clearance, which restrict its effective retinal concentrations (15, 82). Moreover, the development of sustained-release formulations such as thermoresponsive nanoemulgels or liposomal carriers has been explored to overcome these limitations, showing improved corneal permeation and retention, which are critical for effective DR therapy (102, 104). However, these advanced delivery systems require rigorous clinical validation to establish optimal dosing regimens, safety profiles, and long-term efficacy. The lack of standardized dosing guidelines and variability in phytochemical content among natural extracts further complicate dose optimization, making it difficult to compare results across studies or translate preclinical findings into clinical practice (13, 20). Thus, addressing bioavailability and dose standardization is paramount for harnessing the full therapeutic potential of natural antioxidants in DR management.

Another critical challenge in applying natural antioxidant therapies for DR is the considerable inter-individual variability in therapeutic response, which leads to inconsistent efficacy outcomes. This variability arises from genetic, metabolic, and physiological differences among patients, influencing absorption, metabolism, and cellular response to phytochemicals. For example, polymorphisms in antioxidant enzyme genes or variations in the Nrf2 pathway, a master regulator of cellular antioxidant defenses, can modulate the effectiveness of polyphenols and other natural compounds in mitigating oxidative stress in retinal tissues (16, 105). Furthermore, differences in gut microbiota composition affect the biotransformation and bioavailability of orally administered phytochemicals, impacting systemic and ocular levels (86). Clinical trials with antioxidants such as quercetin and ALA have reported variable outcomes, partly attributed to these individual differences, underscoring the need for personalized approaches in antioxidant therapy (15, 59). Additionally, the presence of comorbidities, concurrent medications, and the stage of DR also influence treatment response, complicating the assessment of efficacy in heterogeneous patient populations (13, 40). The complexity of DR pathogenesis, involving multifactorial oxidative, inflammatory, and angiogenic pathways, further necessitates combinational or multi-targeted therapies tailored to individual patient profiles (22, 106). Emerging strategies such as biomarker-guided therapy and advanced drug delivery systems aim to mitigate variability by enhancing targeted delivery and optimizing dosing, but these approaches remain in early stages of development (35, 107). Consequently, overcoming individual variability is essential to achieve consistent and reproducible therapeutic benefits of natural antioxidants in DR treatment.

Nanotechnology-based drug delivery systems have emerged as promising strategies to overcome the bioavailability and dose standardization challenges associated with natural antioxidants. Lipid-polymer hybrid nanoparticles, SNEDDS, NLCs, and polymeric nanoparticles have been developed to enhance the solubility, stability, and ocular penetration of phytochemicals such as curcumin, resveratrol, quercetin, and luteolin. These nanocarriers protect labile compounds from degradation, facilitate controlled release, and enable targeted delivery to retinal tissues through surface functionalization with targeting ligands. For example, TPGS-functionalized lipid-polymer hybrid nanoparticles have demonstrated enhanced oral bioavailability and retinal targeting of luteolin, resulting in superior therapeutic efficacy in diabetic models (49). Similarly, SNEDDS formulations of resveratrol and melatonin have shown improved solubility and stability, facilitating SIRT-1 activation for DR therapy (108). Thermoresponsive nanoemulgels and liposomal carriers have been developed to enhance corneal permeation and retention, which are critical for effective topical ocular delivery (109). However, these advanced delivery systems require rigorous clinical validation to establish optimal dosing regimens, safety profiles, and long-term efficacy. The development of standardized, well-characterized nanoformulations is essential to ensure consistent therapeutic outcomes and facilitate regulatory approval for clinical use.

## 5 Future research directions

### 5.1 Novel drug development

**Figure 5:**
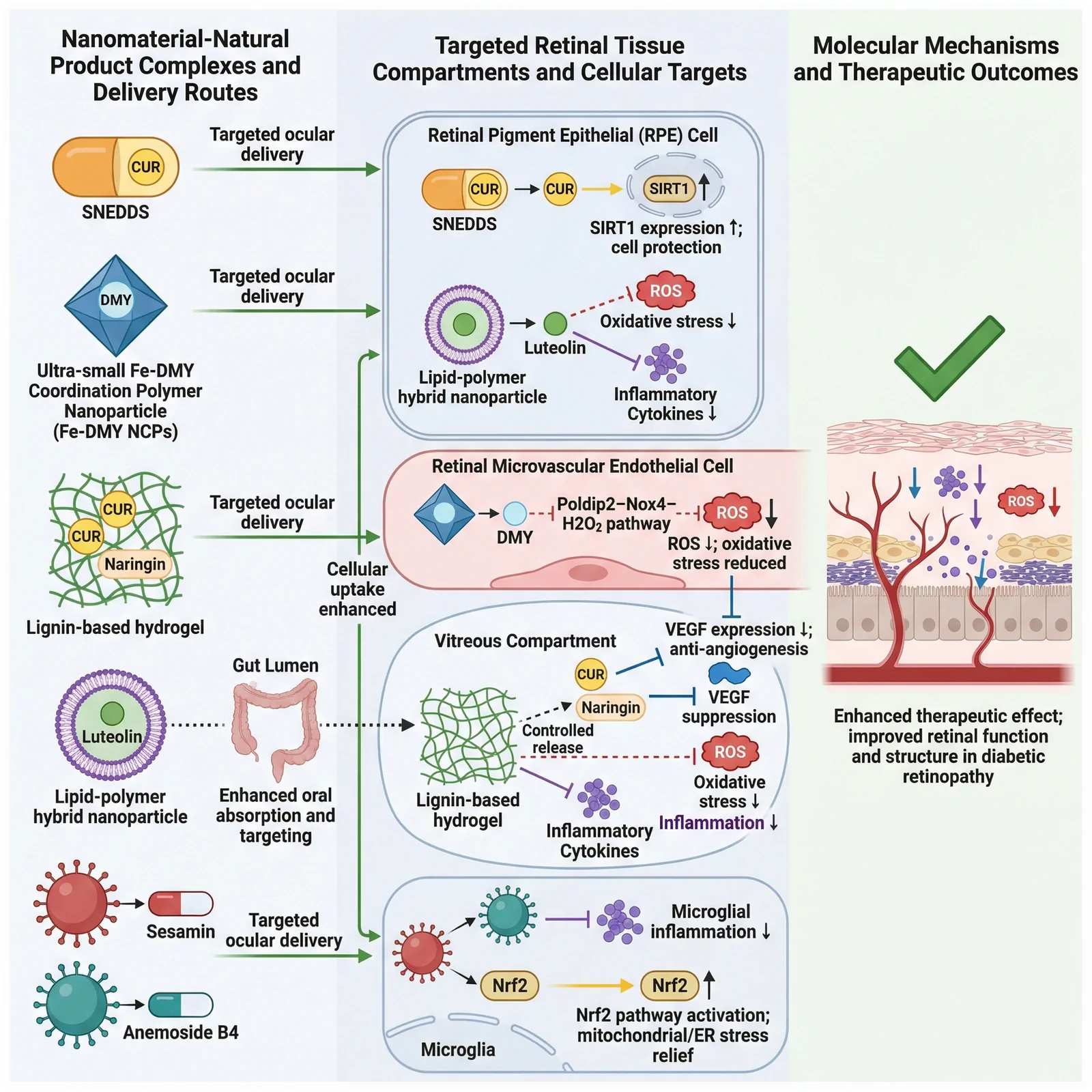
Nanomaterial-based delivery systems for natural antioxidants in diabetic retinopathy therapy. These include SNEDDS, Fe-DMY coordination nanoparticles, lignin-based hydrogels, lipid-polymer hybrid nanoparticles, and other nanocarriers. They enhance ocular targeting, cellular uptake, and oral absorption, delivering curcumin, luteolin, dihydromyricetin, and other compounds to retinal cells (RPE, endothelial cells, microglia) and the vitreous compartment. Mechanistically, they reduce ROS and inflammatory cytokines, activate Nrf2/SIRT1 pathways, inhibit VEGF-mediated angiogenesis, and preserve retinal structure and function, overcoming limitations of native natural products.

The development of novel therapeutic agents for DR increasingly focuses on improving the bioavailability and targeted delivery of natural antioxidants and anti-inflammatory compounds, which often suffer from poor solubility and limited ocular penetration. Nanotechnology-based drug delivery systems have emerged as promising strategies to overcome these limitations by enhancing drug stability, solubility, and tissue-specific accumulation. **Table 2** shows the application of novel nanomaterials loaded with natural antioxidants in the treatment of DR. For instance, curcumin (CUR), a natural polyphenol with potent antioxidant and anti-inflammatory effects, shows therapeutic potential in DR; however, its clinical application is hindered by low aqueous solubility and limited retinal penetration. The formulation of CUR into SNEDDS has demonstrated improved physicochemical stability, mucoadhesive properties, and enhanced intracellular uptake in retinal pigment epithelial cells, resulting in superior antioxidant activity and induction of protective SIRT1 expression compared to free CUR (77). Similarly, the encapsulation of dihydromyricetin (DMY), a natural flavonoid antioxidant, into ultra-small nanoscale coordination polymer particles (Fe-DMY NCPs) has been shown to effectively scavenge ROS, inhibit key oxidative stress pathways such as Poldip2-Nox4-H2O2, and ameliorate vascular leakage and neovascularization in DR models, highlighting the potential of nanocarriers to improve therapeutic efficacy and bioavailability of natural compounds (61). Another innovative approach involves the use of lignin-based hydrogels derived from agricultural waste as drug carriers for natural polyphenolic antioxidants like curcumin and naringenin. These hydrogels exhibit antioxidant and anti-inflammatory properties themselves and provide controlled, non-invasive ocular delivery with demonstrated suppression of VEGF levels in vitreous fluid, addressing both oxidative stress and angiogenesis in DR (110). Lignin-based hydrogels represent an innovative and sustainable approach for ocular drug delivery, serving as both drug carriers and active therapeutic agents due to their intrinsic antioxidant and anti-inflammatory properties. While not strictly nanomaterials in the conventional sense, these hydrogels possess nanoscale structural features and provide controlled, non-invasive ocular delivery with demonstrated suppression of VEGF levels in vitreous fluid. Moreover, lipid-polymer hybrid nanoparticles functionalized with TPGS have been developed to enhance the oral bioavailability of luteolin, a flavonoid with anti-inflammatory and antioxidant activities. The optimized nanoparticles showed prolonged drug release, improved intestinal permeability, and significant attenuation of hyperglycemia-induced retinal oxidative stress, inflammation, and angiogenesis in diabetic models, underscoring the translational potential of nanocarrier systems for systemic delivery of natural therapeutics in DR (49). These advances are complemented by studies on other natural compounds such as sesamin, which inhibits high glucose-induced microglial inflammation and oxidative stress, and plumbagin, which protects retinal structure and function via activation of Nrf2 signaling and mitigation of mitochondrial-endoplasmic reticulum stress, though further formulation improvements are needed to enhance their bioavailability and retinal targeting (44, 111) (**Figure 5**). Collectively, these findings illustrate that integrating nanotechnology with natural antioxidant and anti-inflammatory agents offers a promising avenue to overcome pharmacokinetic barriers, achieve targeted delivery to retinal tissues, and potentiate therapeutic efficacy in DR. Future research should continue to optimize these delivery platforms, investigate their safety and pharmacodynamics in clinical settings, and explore combinatorial strategies to address the multifactorial pathogenesis of DR.

The design of effective nanocarrier systems for natural antioxidant delivery to retinal tissues requires careful consideration of several key parameters, including particle size, surface charge, drug loading capacity, release kinetics, and biocompatibility. Ideal nanocarriers should be small enough (typically <200 nm) to penetrate ocular barriers while avoiding rapid clearance, possess appropriate surface properties to enhance cellular uptake and retinal targeting, and provide sustained drug release to maintain therapeutic concentrations over extended periods. Surface functionalization with targeting ligands such as hyaluronic acid (targeting CD44 receptors), RGD peptides (targeting integrins), or antibodies against specific retinal cell surface markers can further enhance the selectivity and efficacy of these delivery systems (112). Additionally, stimuli-responsive nanocarriers that release their cargo in response to specific pathological cues (e.g., elevated ROS levels in diabetic retinas) offer the potential for on-demand drug release, maximizing therapeutic efficacy while minimizing off-target effects.

Beyond the established nanocarrier systems discussed above, several emerging platforms show promise for natural antioxidant delivery in DR. ROS-responsive nanoparticles have been developed to release their cargo specifically in the oxidative microenvironment of diabetic retinas, as demonstrated by NPSPHE@EOFAZ, which delivers essential oils from Fructus Alpiniae zerumbet to Müller cells, reducing oxidative stress and inflammation in diabetic models (113). Iron-coordinated nanozymes (Fe-Quer NZs) that mimic multiple antioxidant enzymes have been designed to scavenge ROS, alleviate oxidative stress and inflammation, and inhibit angiogenesis and microvascular leakage in early DR (114). Carbon dots derived from medicinal plants (SH-CDs) with good biocompatibility can penetrate ocular tissues, scavenge reactive species, and relieve oxidative stress and inflammation (115). Lignin-based hydrogels derived from agricultural waste represent an innovative and sustainable approach, serving as both drug carriers and active therapeutic agents due to their intrinsic antioxidant and anti-inflammatory properties (110). These emerging platforms highlight the rapid evolution of nanotechnology-based solutions for natural antioxidant delivery and offer exciting possibilities for more effective DR therapy.

### 5.2 Mechanistic studies

The pathogenesis of DR is multifactorial, with oxidative stress playing a central role in its initiation and progression. Hyperglycemia-induced overproduction of ROS disrupts the redox homeostasis in retinal cells, leading to cellular damage, inflammation, and microvascular dysfunction. Natural antioxidant compounds derived from plants and other natural sources have emerged as promising therapeutic agents due to their multifaceted mechanisms targeting oxidative stress and associated pathological pathways in DR. These compounds exert their protective effects through modulation of key signaling cascades, transcription factors, and enzymatic antioxidant defenses, thereby attenuating retinal damage and disease progression. A critical mechanism by which natural antioxidants confer protection in DR involves the activation of the Nrf2 pathway, a master regulator of cellular antioxidant responses. Under hyperglycemic conditions, Nrf2 activity is often suppressed, leading to diminished expression of downstream antioxidant enzymes such as heme oxygenase-1 (HO-1), superoxide dismutase (SOD), catalase (CAT), and glutathione peroxidase (GPX). Several phytochemicals, including carnosol and syringaresinol, have been shown to activate Nrf2 signaling, resulting in enhanced antioxidant enzyme expression and reduced oxidative stress in retinal endothelial cells and animal models of DR (18, 41). The activation of Nrf2 by these compounds involves upstream kinases such as extracellular signal-regulated kinases (ERK1/2), which mediate the nuclear translocation and transcriptional activity of Nrf2, thereby restoring redox balance and protecting retinal microvasculature.

In addition to Nrf2 activation, natural antioxidants modulate other molecular pathways implicated in DR pathogenesis. For example, ALA inhibits NF-κB activity, a pivotal transcription factor driving inflammation and vascular permeability in DR. By suppressing NF-κB, ALA reduces the expression of pro-inflammatory cytokines and matrix metalloproteinases, thereby mitigating retinal inflammation and vascular leakage (15). Similarly, activated growth factor (AGF) derived from platelets has been reported to downregulate multiple oxidative stress and inflammatory mediators, including AGEs, p38 mitogen-activated protein kinase (p38 MAPK), NF-κB, TNF-α, interleukin-1 beta (IL-1β), and VEGF, while enhancing antioxidant enzyme activity such as SOD in diabetic retinal tissue (116) (**Figure 5**). This comprehensive modulation of oxidative and inflammatory pathways underscores the complex network through which natural antioxidants exert their therapeutic effects.

Lipid peroxidation is another critical pathological event in DR, driven by the high content of polyunsaturated fatty acids in the retina. Natural polyphenols like pterostilbene have demonstrated efficacy in reducing lipid peroxidation markers in diabetic models, restoring redox status, and normalizing antioxidant enzyme activities. These effects are linked to the activation of the phosphoinositide 3-kinase (PI3K)/AKT/glycogen synthase kinase 3 beta (GSK3β)/Nrf2 signaling axis, highlighting a sophisticated intracellular mechanism that protects retinal cells from oxidative damage (16, 117, 118). The reduction of lipid peroxidation not only prevents structural retinal damage but also decreases the formation of oxidative stress-derived biomarkers, which may serve as indicators of DR progression and therapeutic response.

Emerging evidence also points to the involvement of long noncoding RNAs (lncRNAs) in regulating antioxidant defense in DR. For instance, MALAT1, a lncRNA upregulated by high glucose, promotes Keap1 expression, which sequesters Nrf2 in the cytoplasm, thereby impairing antioxidant gene transcription. Silencing MALAT1 restores Nrf2 nuclear translocation and enhances antioxidant defenses, suggesting that targeting lncRNAs may represent a novel mechanism to potentiate the efficacy of natural antioxidants in DR (119). Furthermore, the anti-angiogenic effects of natural antioxidants contribute significantly to their therapeutic potential. Compounds such as curcumin and axitinib inhibit VEGF expression and signaling, thereby preventing pathological neovascularization characteristic of proliferative DR. This anti-angiogenic activity often intersects with antioxidant and anti-inflammatory pathways, creating a synergistic effect that stabilizes retinal vasculature and preserves vision (14, 106, 120). For example, syringaresinol suppresses hypoxia-inducible factor-1 alpha (HIF-1α)/VEGF signaling via Nrf2 activation, reducing retinal vascular permeability and endothelial apoptosis (41) (**Figure 5**).

At the cellular level, natural antioxidants also prevent apoptosis of retinal cells by reducing oxidative stress-induced mitochondrial dysfunction and modulating apoptotic signaling pathways. Tocotrienol-rich fractions have been shown to decrease retinal cell apoptosis and preserve retinal morphology and visual function in diabetic rats by enhancing antioxidant enzyme activities and reducing lipid peroxidation (121). Similarly, the antioxidant tetrapeptide Epitalon inhibits hyperglycemia-induced epithelial-mesenchymal transition (EMT) and fibrosis in retinal pigment epithelial cells, promoting wound healing and retinal repair (122).

Nanotechnology-based delivery systems have been explored to enhance the bioavailability and targeted delivery of natural antioxidants to retinal tissues. For instance, ROS-responsive nanoparticles loaded with essential oils from Fructus Alpiniae zerumbet effectively reduce oxidative stress and inflammation in diabetic retinas, demonstrating improved therapeutic outcomes in preclinical models (113). Such advanced delivery platforms may overcome limitations related to poor bioavailability and specificity of natural compounds.

In summary, natural antioxidant products act through a complex network of mechanisms in DR, including activation of the Nrf2 antioxidant pathway, inhibition of NF-κB-mediated inflammation, suppression of lipid peroxidation, modulation of lncRNA-mediated gene regulation, anti-angiogenic effects via VEGF/HIF-1α pathways, and prevention of retinal cell apoptosis. These multifaceted actions collectively contribute to the preservation of retinal structure and function in DR. Continued elucidation of these mechanisms and their interplay will facilitate the development of optimized antioxidant-based therapies and guide clinical translation for effective management of DR. However, challenges such as bioavailability, specificity, and long-term safety remain and warrant further investigation through rigorous preclinical and clinical studies (15, 18, 41, 106, 113, 116, 117, 119–121).

### 5.3 Clinical application promotion

The clinical application of natural antioxidant products in DR management remains an evolving field, with growing preclinical evidence supporting their potential benefits but limited robust clinical validation. Strengthening randomized controlled trials (RCTs) is essential to substantiate the efficacy and safety of these agents in DR patients. Natural compounds such as carotenoids (e.g., lutein, zeaxanthin), polyphenols (e.g., resveratrol, curcumin), and other phytochemicals have demonstrated antioxidative, anti-inflammatory, anti-angiogenic, and neuroprotective effects in various *in vitro* and animal models of DR, suggesting their promise as adjunctive therapies to conventional treatments. For instance, lutein has been shown to reduce oxidative stress and inflammation in retinal tissues, with some clinical trials indicating improvements in visual function and retinal health, although larger, well-designed RCTs are needed to confirm these findings and optimize dosing regimens (88, 123). Similarly, resveratrol exhibits multiple protective mechanisms, including modulation of oxidative stress pathways, suppression of inflammatory cytokines, and inhibition of pathological neovascularization. However, its clinical translation is hampered by poor bioavailability and rapid metabolism, necessitating trials that incorporate novel delivery systems such as nanoformulations to enhance ocular bioavailability and therapeutic efficacy (56). Curcumin, another potent natural antioxidant, has shown promise in preclinical DR models through its anti-inflammatory and antioxidative mechanisms, but clinical trials are limited by its low solubility and bioavailability. Advances in drug delivery platforms, including nanoparticles and cyclodextrin complexes, have improved curcumin’s ocular retention and permeability, warranting further clinical investigation to establish safety and efficacy in DR patients (58, 124). Alpha-lipoic acid, with its mitochondrial cofactor role and antioxidant properties, has demonstrated protective effects against oxidative stress and inflammation in diabetic ocular tissues. Preliminary clinical studies suggest that ALA combined with vitamins and genistein may protect retinal cells and reduce inflammation in pre-retinopathic diabetic patients, but larger RCTs are required to validate these outcomes and define therapeutic protocols (15). Other natural compounds such as crocin and anthocyanins have shown beneficial effects on retinal health through antioxidative and anti-inflammatory pathways, with some clinical evidence indicating improved visual outcomes; nonetheless, comprehensive RCTs with adequate sample sizes and long-term follow-up remain scarce (86, 125). The challenges in clinical translation include suboptimal specificity, low bioavailability, potential toxicity, and variability in natural product formulations. To overcome these barriers, future RCTs should adopt rigorous designs with standardized extracts, optimized dosing, and appropriate delivery systems to enhance bioavailability and target retinal tissues effectively. Additionally, integrating biomarkers of oxidative stress, inflammation, and retinal function into trial endpoints can provide mechanistic insights and facilitate personalized therapy approaches. The use of nanocarriers and sustained-release formulations holds promise to improve therapeutic outcomes and patient compliance. Furthermore, combination therapies incorporating natural antioxidants with existing treatments such as anti-VEGF agents may offer synergistic benefits and should be explored in clinical trials. In conclusion, while preclinical data strongly support the potential of natural antioxidant products in DR management, robust RCTs are imperative to confirm their clinical efficacy and safety, optimize therapeutic regimens, and ultimately translate these promising compounds into standard care for DR (13, 20, 56, 89).

## 6 Conclusion

The exploration of natural antioxidant agents in the treatment of DR represents a promising frontier in addressing the complex pathophysiology of this vision-threatening complication of diabetes. From an expert perspective, the accumulated preclinical and emerging clinical evidence underscores the potential of these compounds to mitigate oxidative stress and inflammatory pathways—two pivotal mechanisms driving retinal damage in DR. The multifaceted antioxidant properties of natural agents not only counteract ROS but also modulate cellular signaling cascades involved in inflammation, apoptosis, and vascular dysfunction, thereby offering a holistic approach to retinal protection.

Balancing the diverse research perspectives, it is clear that while *in vitro* and animal model studies consistently demonstrate beneficial effects of natural antioxidants, translation to human clinical outcomes remains an area requiring rigorous investigation. Variability in study designs, heterogeneity of natural compounds, dosage regimens, and patient populations contribute to the current challenges in establishing definitive clinical efficacy and safety profiles. Moreover, some studies highlight potential limitations such as bioavailability issues and the need for standardized formulations, which are critical factors influencing therapeutic success. From a clinical standpoint, the integration of natural antioxidants into DR management protocols could complement existing treatments, potentially reducing reliance on invasive procedures and pharmacologic agents with adverse effect profiles. However, this integration must be predicated on robust clinical trials that validate efficacy, optimize dosing strategies, and monitor long-term safety. Additionally, understanding the interplay between natural antioxidants and conventional therapies will be essential to avoid adverse interactions and maximize patient outcomes.

A critical advancement that has significantly enhanced the translational potential of natural antioxidants is the integration of nanotechnology-based delivery systems. Nanocarriers effectively address the fundamental limitations of phytochemicals—poor aqueous solubility, low bioavailability, rapid metabolism, and insufficient ocular tissue penetration—that have historically hindered their clinical application. By enhancing solubility, protecting compounds from degradation, facilitating targeted delivery to retinal tissues, and enabling sustained release, nanoformulations amplify the therapeutic efficacy of natural antioxidants and overcome the translational barriers that have limited their clinical utility. The development of smart nanocarriers with stimuli-responsive release, surface functionalization for active targeting, and multifunctional capabilities (e.g., combining antioxidant, anti-inflammatory, and anti-angiogenic effects) represents the next frontier in this field. As nanomedicine platforms continue to evolve and clinical validation studies accumulate, the combination of natural antioxidants with advanced delivery systems holds substantial promise for transforming DR management.

Looking forward, a multidisciplinary approach combining ophthalmology, pharmacology, and nutrition science is vital to advance this field. Future research should prioritize well-designed randomized controlled trials with standardized endpoints, alongside mechanistic studies to elucidate precise molecular targets. Furthermore, personalized medicine approaches considering genetic, metabolic, and lifestyle factors may enhance the therapeutic applicability of natural antioxidants in DR.

In summary, natural antioxidant products hold considerable promise as adjunctive or alternative therapies for DR, particularly through their capacity to attenuate oxidative stress and inflammation. Nonetheless, the transition from experimental promise to clinical reality hinges on comprehensive clinical validation and strategic integration into patient care. As the evidence base strengthens, these agents may well become integral components of a multifaceted strategy to preserve vision and improve quality of life for individuals affected by DR.

## References

[1] Wang X Liu W Zheng X Yang MM . New insights of potential biomarkers in diabetic retinopathy: integrated multi-omic analyses. Front Endocrinol (Lausanne). (2025) 16:1595207. doi: 10.3389/fendo.2025.1595207 40666054 PMC12259430

[2] Mujeeb Rahman KK Nasor M Imran A . Automatic screening of diabetic retinopathy using fundus images and machine learning algorithms. Diagnostics (Basel). (2022) 12. doi: 10.3390/diagnostics12092262 36140666 PMC9497622

[3] Gomułka K Ruta M . The role of inflammation and therapeutic concepts in diabetic retinopathy-a short review. Int J Mol Sci. (2023) 24. doi: 10.3390/ijms24021024 36674535 PMC9864095

[4] Almutairi NM Alahmadi S Alharbi M Gotah S Alharbi M . The association between HbA1c and other biomarkers with the prevalence and severity of diabetic retinopathy. Cureus. (2021) 13:e12520. doi: 10.7759/cureus.12520 33564524 PMC7863112

[5] Serikbaeva A Li Y Ma S Yi D Kazlauskas A . Resilience to diabetic retinopathy. Prog Retin Eye Res. (2024) 101:101271. doi: 10.1016/j.preteyeres.2024.101271 38740254 PMC11262066

[6] Luo Y Li C . Advances in research related to microRNA for diabetic retinopathy. J Diabetes Res. (2024) 2024:8520489. doi: 10.1155/2024/8520489 38375094 PMC10876316

[7] Zhang C Gu L Xie H Liu Y Huang P Zhang J . Glucose transport, transporters and metabolism in diabetic retinopathy. Biochim Biophys Acta Mol Basis Dis. (2024) 1870:166995. doi: 10.1016/j.bbadis.2023.166995 38142757

[8] Rák T Kovács-Valasek A Pöstyéni E Csutak A Gábriel R . Complementary approaches to retinal health focusing on diabetic retinopathy. Cells. (2023) 12. doi: 10.3390/cells12232699 38067127 PMC10705724

[9] Brighenti T Neri G Mazzola M Tomé G Scalfati M Peroni D . Comparative proteomic analysis of human vitreous in rhegmatogenous retinal detachment and diabetic retinopathy reveals a common pathway and potential therapeutic target. Clin Proteomics. (2024) 21:63. doi: 10.1186/s12014-024-09515-3 39609746 PMC11603643

[10] Li J Chen K Li X Zhang X Zhang L Yang Q . Mechanistic insights into the alterations and regulation of the AKT signaling pathway in diabetic retinopathy. Cell Death Discov. (2023) 9:418. doi: 10.1038/s41420-023-01717-2 37978169 PMC10656479

[11] Han X Zhang L Kong L Tong M Shi Z Li XM . Comprehensive metabolic profiling of diabetic retinopathy. Exp Eye Res. (2023) 233:109538. doi: 10.1016/j.exer.2023.109538 37308049

[12] Rampin A Carrabba M Mutoli M Eman CL Testa G Madeddu P . Recent advances in KEAP1/NRF2-targeting strategies by phytochemical antioxidants, nanoparticles, and biocompatible scaffolds for the treatment of diabetic cardiovascular complications. Antioxid Redox Signal. (2022) 36:707–28. doi: 10.1089/ars.2021.0134 35044251

[13] Khidr EG Morad NI Hatem S El-Dessouki AM Mohamed AF El-Shiekh RA . Natural remedies proposed for the management of diabetic retinopathy (DR): diabetic complications. Naunyn Schmiedebergs Arch Pharmacol. (2025) 398:7919–47. doi: 10.1007/s00210-025-03866-w 39954069 PMC12263737

[14] Amini S Sahebkar A Dehghani A Iraj B Rezaeian-Ramsheh A Askari G . The effect of curcumin-piperine on cardiometabolic, inflammatory and oxidative stress factors and macular vascular density in optical coherence tomography angiography (OCTA) in patients with non-proliferative diabetic retinopathy: study protocol for a randomized, double-blind controlled trial. Avicenna J Phytomed. (2023) 13:153–64. doi: 10.22038/ajp.2022.21512 37333470 PMC10274315

[15] Ajith TA . Alpha-lipoic acid: a possible pharmacological agent for treating dry eye disease and retinopathy in diabetes. Clin Exp Pharmacol Physiol. (2020) 47:1883–90. doi: 10.1111/1440-1681.13373 32621549

[16] Millán I Desco MDC Torres-Cuevas I Pérez S Pulido I Mena-Mollá S . Pterostilbene prevents early diabetic retinopathy alterations in a rabbit experimental model. Nutrients. (2019) 12. doi: 10.3390/nu12010082 31892189 PMC7019414

[17] Gowd V Xiao J Wang M Chen F Cheng KW . Multi-mechanistic antidiabetic potential of astaxanthin: an update on preclinical and clinical evidence. Mol Nutr Food Res. (2021) 65:e2100252. doi: 10.1002/mnfr.202100252 34636497

[18] D'Agata V D'Amico AG Maugeri G Bucolo C Rossi S Giunta S . Carnosol attenuates high glucose damage in human retinal endothelial cells through regulation of ERK/Nrf2/HO-1 pathway. J Asian Nat Prod Res. (2023) 25:783–95. doi: 10.1080/10286020.2022.2137022 36300534

[19] Ashraf Mahmoud Khashaba N Abdel Hafez SMN Abdelzaher WY Ahmed Rifaai R Abdel Majeed NAM . Therapeutic effects of selenium nanoparticles versus selenium on experimentally induced diabetic retinopathy via modulation of TLR4 / NF-(k)B P65 / VEGF / connexin 43 signaling. Tissue Barriers. (2025) 13:2491911. doi: 10.1080/21688370.2025.2491911 40247438 PMC12667640

[20] Zhao Y Chen Y Yan N . The role of natural products in diabetic retinopathy. Biomedicines. (2024) 12. doi: 10.3390/biomedicines12061138 38927345 PMC11200400

[21] Zhao D Wang C Jiang Z Xu Y Xia Y Zhang P . Nanomaterial-enabled delivery of plant-derived bioactive metabolites for diabetic retinopathy: from evidence appraisal to preclinical translation. Front Pharmacol. (2026) 17:1809139. doi: 10.3389/fphar.2026.1809139 42434121 PMC13349831

[22] Gómez-Jiménez V Burggraaf-Sánchez de Las Matas R Ortega ÁL . Modulation of oxidative stress in diabetic retinopathy: therapeutic role of natural polyphenols. Antioxidants (Basel). (2025) 14. doi: 10.3390/antiox14070875 40722979 PMC12292083

[23] Jin Y Arroo R . The protective effects of flavonoids and carotenoids against diabetic complications-a review of *in vivo* evidence. Front Nutr. (2023) 10:1020950. doi: 10.3389/fnut.2023.1020950 37032781 PMC10080163

[24] Liu C Wang X Liu M Cao X . Molecular insights into carotenoid-based interventions for diabetic retinopathy. Nutr Bull. (2025) 50:421–35. doi: 10.1111/nbu.70015 40629811

[25] Singh AK Kumar P Rajput VD Mishra SK Tiwari KN Singh AK . Phytochemicals, antioxidant, anti-inflammatory studies, and identification of bioactive compounds using GC-MS of ethanolic novel polyherbal extract. Appl Biochem Biotechnol. (2023) 195:4447–68. doi: 10.1007/s12010-023-04363-7 36701094

[26] Amin R Shariff MA Purwanita P Saleh MI . Efficacy of Sambiloto extracts, Andrographis paniculate, (Burm. F) in inhibiting diabetic retinopathy progression: an *in vivo* study. Rep Biochem Mol Biol. (2022) 11:457–64. doi: 10.52547/rbmb.11.3.457 36718307 PMC9883034

[27] Peng J Abdulla R Liu X He F Xin X Aisa HA . Polyphenol-rich extract of Apocynum venetum L. leaves protects human retinal pigment epithelial cells against high glucose-induced damage through polyol pathway and autophagy. Nutrients. (2024) 16. doi: 10.3390/nu16172944 39275261 PMC11397065

[28] Ma H Li J . The ginger extract could improve diabetic retinopathy by inhibiting the expression of e/iNOS and G6PDH, apoptosis, inflammation, and angiogenesis. J Food Biochem. (2022) 46:e14084. doi: 10.1111/jfbc.14084 35060143

[29] Sukboon P Phumsuay R Promkum C Thiyajai P Sukprasansap M Muangnoi C . Mulberry extract mitigates glucose-induced oxidative injury in differentiated ARPE-19 cells by enhancing antioxidant defense: implications for diabetic retinopathy. Food Sci Nutr. (2025) 13:e70180. doi: 10.1002/fsn3.70180 40330202 PMC12052892

[30] Thomas JO Idowu IM . Phytochemicals in Ginkgo biloba for age-related eye disease - a review. J Diet Suppl. (2025) 22:907–38. doi: 10.1080/19390211.2025.2560365 40947728

[31] Ho KL Yong PH Wang CW Kuppusamy UR Ngo CT Massawe F . Peperomia pellucida (L.) Kunth and eye diseases: a review on phytochemistry, pharmacology and toxicology. J Integr Med. (2022) 20:292–304. doi: 10.1016/j.joim.2022.02.002 35153134

[32] Ho KL Yong PH Wang CW Lim SH Kuppusamy UR Arumugam B . *In vitro* anti-inflammatory activity and molecular docking of Peperomia pellucida (L.) Kunth extract via the NF-κB and PPAR-γ signalling in human retinal pigment epithelial cells. Bioorg Chem. (2024) 153:107969. doi: 10.1016/j.bioorg.2024.107969 39561439

[33] Alagarsamy V Sulthana MT Solomon VR Satishchandra A Kulkarni VS Narendhar B . Identification of potential inhibitors from medicinal plant-based phytochemicals for the influential C4 target of diabetic retinopathy by molecular docking studies. Curr Pharm Des. (2025) 31:307–19. doi: 10.2174/0113816128297758240723104452 39129155

[34] Gui S Tang W Huang Z Wang X Gui S Gao X . Ultrasmall coordination polymer nanodots Fe‐Quer nanozymes for preventing and delaying the development and progression of diabetic retinopathy. Adv Funct Mater. (2023) 33. doi: 10.1002/adfm.20230026142587375

[35] Pu N Li S Wu H Zhao N Wang K Wei D . Beacon of hope for age-related retinopathy: antioxidative mechanisms and pre-clinical trials of quercetin therapy. Antioxidants (Basel). (2025) 14. doi: 10.3390/antiox14050561 40427443 PMC12108410

[36] Zhang Y Li M Liu H Fan Y Liu HH . The application of procyanidins in diabetes and its complications: a review of preclinical studies. Front Pharmacol. (2025) 16:1532246. doi: 10.3389/fphar.2025.1532246 39995417 PMC11847907

[37] Kanwugu ON Glukhareva TV Danilova IG Kovaleva EG . Natural antioxidants in diabetes treatment and management: prospects of astaxanthin. Crit Rev Food Sci Nutr. (2022) 62:5005–28. doi: 10.1080/10408398.2021.1881434 33591215

[38] Tang Y Fang C Shi J Chen H Chen X Yao X . Antioxidant potential of chlorogenic acid in age-related eye diseases. Pharmacol Res Perspect. (2024) 12:e1162. doi: 10.1002/prp2.1162 38189160 PMC10772849

[39] He W Tang P Lv H . Targeting oxidative stress in diabetic retinopathy: mechanisms, pathology, and novel treatment approaches. Front Immunol. (2025) 16:1571576. doi: 10.3389/fimmu.2025.1571576 40589740 PMC12207546

[40] Dilworth L Stennett D Facey A Omoruyi F Mohansingh S Omoruyi FO . Diabetes and the associated complications: the role of antioxidants in diabetes therapy and care. BioMed Pharmacother. (2024) 181:117641. doi: 10.1016/j.biopha.2024.117641 39541789

[41] Liu C Cheng T Wang Y Li G Wang Y Tian W . Syringaresinol alleviates early diabetic retinopathy by downregulating HIF-1α/VEGF via activating Nrf2 antioxidant pathway. Mol Nutr Food Res. (2024) 68:e2200771. doi: 10.1002/mnfr.202200771 38356045

[42] Xie T Chen X Chen W Huang S Peng X Tian L . Curcumin is a potential adjuvant to alleviates diabetic retinal injury via reducing oxidative stress and maintaining Nrf2 pathway homeostasis. Front Pharmacol. (2021) 12:796565. doi: 10.3389/fphar.2021.796565 34955862 PMC8702852

[43] Koushki M Farahani M Yekta RF Frazizadeh N Bahari P Parsamanesh N . Potential role of resveratrol in prevention and therapy of diabetic complications: a critical review. Food Nutr Res. (2024) 68. doi: 10.29219/fnr.v68.9731 38716357 PMC11075469

[44] Zhang J Li L Xiu F . Sesamin suppresses high glucose-induced microglial inflammation in the retina *in vitro* and *in vivo*. J Neurophysiol. (2022) 127:405–11. doi: 10.1152/jn.00466.2021 35020533

[45] Nirbhavane P Magar AG Nimse M Kale SS Chalikwar SS Moravkar KK . Design and evaluation of curcumin, naringenin, and alpha-lactalbumin encapsulated nanostructured lipid carriers for targeted ocular drug delivery in diabetic retinopathy. Exp Eye Res. (2025) 259:110520. doi: 10.1016/j.exer.2025.110520 40675436

[46] Fang J Bai W Yang L . Astaxanthin inhibits oxidative stress and apoptosis in diabetic retinopathy. Acta Histochem. (2023) 125:152069. doi: 10.1016/j.acthis.2023.152069 37343496

[47] Tu Y Li L Zhu L Guo Y Du S Zhang Y . Geniposide attenuates hyperglycemia-induced oxidative stress and inflammation by activating the Nrf2 signaling pathway in experimental diabetic retinopathy. Oxid Med Cell Longev. (2021) 2021:9247947. doi: 10.1155/2021/9247947 34938383 PMC8687848

[48] Lv XM Li N Chen LW Sun C . A comprehensive and systematic review on resveratrol supplementation as a promising candidate for the retinal disease: a focus on mechanisms of action from preclinical studies. Front Pharmacol. (2025) 16:1615910. doi: 10.3389/fphar.2025.1615910 40717982 PMC12289659

[49] Abdelmonem M Al-Mokaddem AK Zakaria MY . TPGS-functionalized nanocarriers with improved flavonoid oral bioavailability and therapeutic action: pharmacokinetic and mechanistic insights in diabetes-induced retinopathy. Eur J Pharm Biopharm. (2025) 216:114851. doi: 10.1016/j.ejpb.2025.114851 40902834

[50] Xu Y Yu J . Allicin mitigates diabetic retinopathy in rats by activating phosphatase and tensin homolog-induced kinase 1/Parkin-mitophagy and inhibiting oxidative stress-mediated NOD-like receptor family pyrin domain containing 3 inflammasome. J Physiol Investig. (2024) 67:215–24. doi: 10.4103/ejpi.EJPI-D-24-00039 39206781

[51] Shi Q Wang J Cheng Y Dong X Zhang M Pei C . Palbinone alleviates diabetic retinopathy in STZ-induced rats by inhibiting NLRP3 inflammatory activity. J Biochem Mol Toxicol. (2020) 34:e22489. doi: 10.1002/jbt.22489 32202043

[52] Zhang Y Gao Z Gao X Yuan Z Ma T Li G . Tilianin protects diabetic retina through the modulation of Nrf2/TXNIP/NLRP3 inflammasome pathways. J Environ Pathol Toxicol Oncol. (2020) 39:89–99. doi: 10.1615/JEnvironPatholToxicolOncol.2020032544 32479015

[53] Tu Y Song E Wang Z Ji N Zhu L Wang K . Melatonin attenuates oxidative stress and inflammation of Müller cells in diabetic retinopathy via activating the Sirt1 pathway. BioMed Pharmacother. (2021) 137:111274. doi: 10.1016/j.biopha.2021.111274 33517190

[54] Huang S Xu D Zhang L Hao L Jia Y Zhang X . Therapeutic effects of curcumin liposomes and nanocrystals on inflammatory osteolysis: *In vitro* and *in vivo* comparative study. Pharmacol Res. (2023) 192:106778. doi: 10.1016/j.phrs.2023.106778 37094714

[55] Margheri F Anceschi C Frediani E Marzoppi A Vasarri M Degl'Innocenti D . Posidonia oceanica extract inhibits VEGF-induced angiogenic and oxidative responses in human endothelial colony-forming cells. J Xenobiot. (2025) 15. doi: 10.3390/jox15050153 40981364 PMC12452316

[56] Kaštelan S Konjevoda S Sarić A Urlić I Lovrić I Čanović S . Resveratrol as a novel therapeutic approach for diabetic retinopathy: Molecular mechanisms, clinical potential, and future challenges. Molecules. (2025) 30. doi: 10.3390/molecules30153262 40807437 PMC12348093

[57] Cheng YW Huang YC Chang KF Huang XF Sheu GT Tsai NM . Protective effect of curcumin on the tight junction integrity and cellular senescence in human retinal pigment epithelium of early diabetic retinopathy. J Physiol Investig. (2024) 67:107–17. doi: 10.4103/ejpi.EJPI-D-23-00035 38857204

[58] Ribeiro A Oliveira D Cabral-Marques H . Curcumin in ophthalmology: Mechanisms, challenges, and emerging opportunities. Molecules. (2025) 30. doi: 10.3390/molecules30030457 39942561 PMC11820683

[59] Saikia L Barbhuiya SAA Saikia K Kalita P Dutta PP . Therapeutic potential of quercetin in diabetic neuropathy and retinopathy: Exploring molecular mechanisms. Curr Top Med Chem. (2024) 24:2351–61. doi: 10.2174/0115680266330678240821060623 39253913

[60] Zeppieri M Gagliano C D'Esposito F Musa M Gattazzo I Zanella MS . Eicosapentaenoic acid (EPA) and docosahexaenoic acid (DHA): A targeted antioxidant strategy to counter oxidative stress in retinopathy. Antioxidants (Basel). (2024) 14. doi: 10.3390/antiox14010006 39857340 PMC11759855

[61] Gui SY Wang XC Huang ZH Li MM Wang JH Gui SY . Nanoscale coordination polymer Fe-DMY downregulating Poldip2-Nox4-H(2)O(2) pathway and alleviating diabetic retinopathy. J Pharm Anal. (2023) 13:1326–45. doi: 10.1016/j.jpha.2023.05.002 38174114 PMC10759264

[62] Apaolaza PS Busch M Asin-Prieto E Peynshaert K Rathod R Remaut K . Hyaluronic acid coating of gold nanoparticles for intraocular drug delivery: Evaluation of the surface properties and effect on their distribution. Exp Eye Res. (2020) 198:108151. doi: 10.1016/j.exer.2020.108151 32721426

[63] Xie Z Yang Z Tian D Chen Y . Unlocking the potential of GLP-1 receptor agonists in ocular therapeutics: from molecular pathways to clinical impact. Front Pharmacol. (2025) 16:1618079. doi: 10.3389/fphar.2025.1618079 40777992 PMC12328412

[64] Wang S Du S Wang W Zhang F . Therapeutic investigation of quercetin nanomedicine in a zebrafish model of diabetic retinopathy. BioMed Pharmacother. (2020) 130:110573. doi: 10.1016/j.biopha.2020.110573 32745912

[65] Chai GR Liu S Yang HW Chen XL . Quercetin protects against diabetic retinopathy in rats by inducing heme oxygenase-1 expression. Neural Regener Res. (2021) 16:1344–50. doi: 10.4103/1673-5374.301027 33318415 PMC8284280

[66] Li R Chen L Yao GM Yan HL Wang L . Effects of quercetin on diabetic retinopathy and its association with NLRP3 inflammasome and autophagy. Int J Ophthalmol. (2021) 14:42–9. doi: 10.18240/ijo.2021.01.06 33469482 PMC7790678

[67] Li H Li B Zheng Y . Exploring the mechanism of action compound-Xueshuantong capsule in diabetic retinopathy treatment based on network pharmacology. Evid Based Complement Alternat Med. (2020) 2020:8467046. doi: 10.1155/2020/8467046 32963574 PMC7499338

[68] Tian R Jin Z Zhou L Zeng XP Lu N . Quercetin attenuated myeloperoxidase-dependent HOCl generation and endothelial dysfunction in diabetic vasculature. J Agric Food Chem. (2021) 69:404–13. doi: 10.1021/acs.jafc.0c06335 33395297

[69] Medoro A Davinelli S Scuderi L Scuderi G Scapagnini G Fragiotta S . Targeting senescence, oxidative stress, and inflammation: Quercetin-based strategies for ocular diseases in older adults. Clin Interv Aging. (2025) 20:791–813. doi: 10.2147/cia.S516946 40503074 PMC12155388

[70] Wu J Lv T Liu Y Liu Y Han Y Liu X . The role of quercetin in NLRP3-associated inflammation. Inflammopharmacology. (2024) 32:3585–610. doi: 10.1007/s10787-024-01566-0 39306817

[71] Abdelkader NF Eitah HE Maklad YA Gamaleldin AA Badawi MA Kenawy SA . New combination therapy of gliclazide and quercetin for protection against STZ-induced diabetic rats. Life Sci. (2020) 247:117458. doi: 10.1016/j.lfs.2020.117458 32092333

[72] Sah MK Gautam B Pokhrel KP Ghani L Bhattarai A . Quantification of the quercetin nanoemulsion technique using various parameters. Molecules. (2023) 28. doi: 10.3390/molecules28062540 36985511 PMC10052722

[73] Yu Y Xu S Yu S Li J Tan G Li S . A hybrid genipin-cross-linked hydrogel/nanostructured lipid carrier for ocular drug delivery: Cellular, ex vivo, and *in vivo* evaluation. ACS Biomater Sci Eng. (2020) 6:1543–52. doi: 10.1021/acsbiomaterials.9b01800 33455373

[74] Amini S Dehghani A Sahebkar A Iraj B Rezaeian-Ramsheh A Askari G . The efficacy of curcumin-piperine supplementation in patients with nonproliferative diabetic retinopathy: An optical coherence tomography angiography-based randomized controlled trial. J Res Med Sci. (2024) 29:64. doi: 10.4103/jrms.jrms_174_24 39744187 PMC11691056

[75] Filippelli M Campagna G Vito P Zotti T Ventre L Rinaldi M . Anti-inflammatory effect of curcumin, homotaurine, and vitamin D3 on human vitreous in patients with diabetic retinopathy. Front Neurol. (2020) 11:592274. doi: 10.3389/fneur.2020.592274 33633656 PMC7901953

[76] Hussain Y Khan H Alotaibi G Khan F Alam W Aschner M . How curcumin targets inflammatory mediators in diabetes: Therapeutic insights and possible solutions. Molecules. (2022) 27. doi: 10.3390/molecules27134058 35807304 PMC9268477

[77] Zingale E Masuzzo S Lajunen T Reinisalo M Rautio J Consoli V . Protective role and enhanced intracellular uptake of curcumin in retinal cells using self-emulsifying drug delivery systems (SNEDDS). Pharm (Basel). (2025) 18. doi: 10.3390/ph18020265 40006077 PMC11859040

[78] Ganugula R Arora M Dwivedi S Chandrashekar DS Varambally S Scott EM . Systemic anti-inflammatory therapy aided by curcumin-laden double-headed nanoparticles combined with injectable long-acting insulin in a rodent model of diabetes eye disease. ACS Nano. (2023) 17:6857–74. doi: 10.1021/acsnano.3c00535 36951721

[79] Ameer N Hanif M Abbas G Azeem M Mahmood K Shahwar D . Treatment of inflammatory bowel disease by using curcumin-containing self-microemulsifying delivery system: Macroscopic and microscopic analysis. Pharmaceutics. (2024) 16. doi: 10.3390/pharmaceutics16111406 39598530 PMC11597465

[80] Jeffrey S Samraj PI Raj BS . The role of alpha-lipoic acid supplementation in the prevention of diabetes complications: A comprehensive review of clinical trials. Curr Diabetes Rev. (2021) 17:e011821190404. doi: 10.2174/1573399817666210118145550 33461470

[81] Oraebosi MI Olurishe TO Anafi SB Bisalla M . Chronopharmacology of the alpha-lipoic acid/nifedipine/glimepiride combination in the amelioration of retinopathy in rats. Chronobiol Int. (2021) 38:443–50. doi: 10.1080/07420528.2020.1866004 33356611

[82] Bierbrauer KL Comini LR Leonhard V Escobar Manzanelli MA Castelli G Farfán S . Eudragit films as carriers of lipoic acid for transcorneal permeability. Polymers (Basel). (2023) 15. doi: 10.3390/polym15071793 37050407 PMC10097161

[83] Mateo Orobia AJ Benítez Del Castillo JM Calonge M Baudouin C Labetoulle M . A narrative literature review about alpha-lipoic acid role in dry eye and ocular surface disease. Acta Ophthalmol. (2025) 103:e346–63. doi: 10.1111/aos.17486 40207422 PMC12340195

[84] Shi C Wang P Airen S Brown C Liu Z Townsend JH . Nutritional and medical food therapies for diabetic retinopathy. Eye Vis (Lond). (2020) 7:33. doi: 10.1186/s40662-020-00199-y 32582807 PMC7310218

[85] Mosallaei N Malaekeh-Nikouei A Sarraf Shirazi S Behmadi J Malaekeh-Nikouei B . A comprehensive review on alpha-lipoic acid delivery by nanoparticles. Bioimpacts. (2024) 14:30136. doi: 10.34172/bi.2024.30136 39493899 PMC11530970

[86] Mistry PS Chorawala MR Sivamaruthi BS Prajapati BG Kumar A Chaiyasut C . The role of dietary anthocyanins for managing diabetes mellitus-associated complications. Curr Diabetes Rev. (2025) 21:e15733998322754. doi: 10.2174/0115733998322754240802063730 39136514 PMC12174903

[87] Johra FT Bepari AK Bristy AT Reza HM . A mechanistic review of β-carotene, lutein, and zeaxanthin in eye health and disease. Antioxidants (Basel). (2020) 9. doi: 10.3390/antiox9111046 33114699 PMC7692753

[88] Akkewar AS Mishra KA Kamble MG Kumar S Dey J Sethi KK . A mechanistic review on growing multiple therapeutic applications of lutein and its global market research. Phytother Res. (2024) 38:3190–217. doi: 10.1002/ptr.8197 38634408

[89] Ye X Fung NSK Lam WC Lo ACY . Nutraceuticals for diabetic retinopathy: Recent advances and novel delivery systems. Nutrients. (2024) 16. doi: 10.3390/nu16111715 38892648 PMC11174689

[90] D'Angelo A Lixi F Vitiello L Gagliardi V Pellegrino A Giannaccare G . The role of diet and oral supplementation for the management of diabetic retinopathy and diabetic macular edema: A narrative review. BioMed Res Int. (2025) 2025:6654976. doi: 10.1155/bmri/6654976 40041571 PMC11876532

[91] Karti O Saatci AO . Fenofibrate and diabetic retinopathy. Med Hypothesis Discov Innov Ophthalmol. (2024) 13:35–43. doi: 10.51329/mehdiophthal1492 38978827 PMC11227662

[92] Liu M Lim ST Song W Coffman TM Wang X . Beyond lipids: fenofibrate in diabetic retinopathy and nephropathy. Trends Pharmacol Sci. (2025). doi: 10.1016/j.tips.2025.07.014 40835560

[93] Liu X Chang Y Li Y Liu Y Song W Lu J . Oxidative stress and retinopathy: evidence from epidemiological studies. J Transl Med. (2025) 23:94. doi: 10.1186/s12967-025-06110-4 39838377 PMC11748554

[94] Mehta N Akram M Singh YP . The impact of zinc supplementation on hyperglycemia and complications of type 2 diabetes mellitus. Cureus. (2024) 16:e73473. doi: 10.7759/cureus.73473 39664139 PMC11634216

[95] Callan A Jha S Valdez L Tsin A . Cellular and molecular mechanisms of neuronal degeneration in early-stage diabetic retinopathy. Curr Vasc Pharmacol. (2024) 22:301–15. doi: 10.2174/0115701611272737240426050930 38693745 PMC13237750

[96] Zhang LY Zhang TY Zheng YJ Li JX Chen HR Cao J . Mechanisms and applications of natural plant ingredients in modulating amino acid metabolism for the improvement of diabetic retinopathy: a review. Front Endocrinol (Lausanne). (2026) 17:1745507. doi: 10.3389/fendo.2026.1745507 41767379 PMC12935674

[97] Mahoney SE Loprinzi PD . Influence of flavonoid-rich fruit and vegetable intake on diabetic retinopathy and diabetes-related biomarkers. J Diabetes Complications. (2014) 28:767–71. doi: 10.1016/j.jdiacomp.2014.06.011 25055729

[98] Amini S Dehghani A Sahebkar A Iraj B Rezaeian-Ramsheh A Askari G . The effect of curcumin plus piperine on mental health status, sleep quality, and anthropometric indices in patients with non-proliferative diabetic retinopathy: A randomized double-blind placebo-controlled trial. Int J Prev Med. (2026) 17:14. doi: 10.4103/ijpvm.ijpvm_276_23 41924225 PMC13038278

[99] Zhang PC Wu CR Wang ZL Wang LY Han Y Sun SL . Effect of lutein supplementation on visual function in nonproliferative diabetic retinopathy. Asia Pac J Clin Nutr. (2017) 26:406–11. doi: 10.6133/apjcn.032016.13 28429904

[100] Alfonso-Muñoz EA Burggraaf-Sánchez de Las Matas R Mataix Boronat J Molina Martín JC Desco C . Role of oral antioxidant supplementation in the current management of diabetic retinopathy. Int J Mol Sci. (2021) 22. doi: 10.3390/ijms22084020 33924714 PMC8069935

[101] Demidova TY Trakhtenberg YA . Alpha-lipoic acid in non-proliferative diabetic retinopathy treatment. Endocrinol Res Centre. (2011) 14:82–6. doi: 10.14341/2072-0351-5823

[102] Singh S Kushwaha P Gupta S . In situ forming nanoemulgel for diabetic retinopathy: Development, characterization, and *in vitro* efficacy assessment. Drug Res (Stuttg). (2025) 75:100–13. doi: 10.1055/a-2517-4967 39919823

[103] Biju BK Andavar M . Resveratrol: potential therapeutic effects on ocular health. Inflammopharmacology. (2025) 33:5757–68. doi: 10.1007/s10787-025-01947-z 40987979

[104] Parashar R Suresh PK . Emerging liposomal therapies for diabetic retinopathy: a review of novel targeting approaches and advances in retinal health outcomes. Drug Delivery. (2025) 32:2509973. doi: 10.1080/10717544.2025.2509973 40438922 PMC12123966

[105] Ebrahimi R Mohammadpour A Medoro A Davinelli S Saso L Miroliaei M . Exploring the links between polyphenols, Nrf2, and diabetes: A review. BioMed Pharmacother. (2025) 186:118020. doi: 10.1016/j.biopha.2025.118020 40168723

[106] Khazeei Tabari MA Mirjalili R Khoshhal H Shokouh E Khandan M Hasheminasabgorji E . Nature against diabetic retinopathy: A review on antiangiogenic, antioxidant, and anti-inflammatory phytochemicals. Evid Based Complement Alternat Med. (2022) 2022:4708527. doi: 10.1155/2022/4708527 35310030 PMC8926515

[107] Diress M Wagle SR Lim P Foster T Kovacevic B Ionescu CM . Advanced drug delivery strategies for diabetic retinopathy: current therapeutic advancement, and delivery methods overcoming barriers, and experimental modalities. Expert Opin Drug Delivery. (2024) 21:1859–77. doi: 10.1080/17425247.2024.2431577 39557623

[108] Zingale E Bonaccorso A D'Amico AG Lombardo R D'Agata V Rautio J . Formulating resveratrol and melatonin self-nanoemulsifying drug delivery systems (SNEDDS) for ocular administration using design of experiments. Pharmaceutics. (2024) 16. doi: 10.3390/pharmaceutics16010125 38258134 PMC10819881

[109] Verma P Singh RK Wadhwa A Prabhakar B Yadav KS . Bimatoprost-loaded lipidic nanoformulation development using quality by design: liposomes versus solid lipid nanoparticles in intraocular pressure reduction. Nanomedicine (Lond). (2023). doi: 10.2217/nnm-2023-0141 37991004

[110] Nirbhavane P Kale SS Kumar S Athare T Magar AG Chalikwar SS . Cotton stalk derived lignin-based hydrogel and its therapeutic utility in diabetic retinopathy. Biochem Biophys Res Commun. (2025) 775:152123. doi: 10.1016/j.bbrc.2025.152123 40466364

[111] Catalani E Del Quondam S Brunetti K Cherubini A Bongiorni S Taddei AR . Neuroprotective role of plumbagin on eye damage induced by high-sucrose diet in adult fruit fly Drosophila melanogaster. BioMed Pharmacother. (2023) 166:115298. doi: 10.1016/j.biopha.2023.115298 37597318

[112] Zhang F Li R Yan M Li Q Li Y Wu X . Ultra-small nanocomplexes based on polyvinylpyrrolidone K-17PF: A potential nanoplatform for the ocular delivery of kaempferol. Eur J Pharm Sci. (2020) 147:105289. doi: 10.1016/j.ejps.2020.105289 32145428

[113] Li J Liu Y Geng K Lu X Shen X Guo Q . ROS-responsive nanoparticles with antioxidative effect for the treatment of diabetic retinopathy. J Biomater Sci Polym Ed. (2025) 36:440–61. doi: 10.1080/09205063.2024.2406628 39316729

[114] Gui S Tang W Huang Z Wang X Gui S Gao X . Ultrasmall coordination polymer nanodots Fe-Quer nanozymes for preventing and delaying the development and progression of diabetic retinopathy. Adv Funct Mater. (2023) 33:2300261. doi: 10.1002/adfm.20230026142587375

[115] Wang J Zhao Y Yang X Li C Liu K Ma C . Delayed progression of diabetic cataractogenesis and retinopathy by topical administration of highly biocompatible carbon dots derived from Scutellaria barbata and Herba hedyotis diffusae. Chem Eng J. (2025) 525:170369. doi: 10.1016/j.cej.2025.17036942574925

[116] Amin R Hidayat R Maritska Z Putri TW . Activated growth factor from platelets as treatment for diabetic retinopathy through antioxidant-oxidative stress pathway. Diabetes Metab Syndr Obes. (2025) 18:305–13. doi: 10.2147/dmso.S490055 39906695 PMC11793107

[117] Torres-Cuevas I Millán I Asensi M Vento M Oger C Galano JM . Analysis of lipid peroxidation by UPLC-MS/MS and retinoprotective effects of the natural polyphenol pterostilbene. Antioxidants (Basel). (2021) 10. doi: 10.3390/antiox10020168 33498744 PMC7912566

[118] Burggraaf-Sánchez de Las Matas R Torres-Cuevas I Millán I Desco MDC Oblaré-Delgado C Asensi M . Potential of pterostilbene as an antioxidant therapy for delaying retinal damage in diabetic retinopathy. Antioxidants (Basel). (2025) 14. doi: 10.3390/antiox14030244 40227230 PMC11939822

[119] Radhakrishnan R Kowluru RA . Long noncoding RNA MALAT1 and regulation of the antioxidant defense system in diabetic retinopathy. Diabetes. (2021) 70:227–39. doi: 10.2337/db20-0375 33051272 PMC7881848

[120] Lazzara F Conti F Sasmal PK Alikunju S Rossi S Drago F . Anti-angiogenic and antioxidant effects of axitinib in human retinal endothelial cells: implications in diabetic retinopathy. Front Pharmacol. (2024) 15:1415846. doi: 10.3389/fphar.2024.1415846 38953109 PMC11215076

[121] Sadikan MZ Nasir NAA Iezhitsa I Agarwal R . Antioxidant and anti-apoptotic effects of tocotrienol-rich fraction against streptozotocin-induced diabetic retinopathy in rats. BioMed Pharmacother. (2022) 153:113533. doi: 10.1016/j.biopha.2022.113533 36076612

[122] Gatta M Dovizio M Milillo C Ruggieri AG Sallese M Antonucci I . The antioxidant tetrapeptide epitalon enhances delayed wound healing in an *in vitro* model of diabetic retinopathy. Stem Cell Rev Rep. (2025) 21:1822–34. doi: 10.1007/s12015-025-10911-x 40493162 PMC12356729

[123] Ikonne EU Ikpeazu VO Ugbogu EA . The potential health benefits of dietary natural plant products in age related eye diseases. Heliyon. (2020) 6:e04408. doi: 10.1016/j.heliyon.2020.e04408 32685729 PMC7355812

[124] Kao YW Hsu SK Chen JY Lin IL Chen KJ Lee PY . Curcumin metabolite tetrahydrocurcumin in the treatment of eye diseases. Int J Mol Sci. (2020) 22. doi: 10.3390/ijms22010212 33379248 PMC7795090

[125] Heydari M Zare M Badie MR Watson RR Talebnejad MR Afarid M . Crocin as a vision supplement. Clin Exp Optom. (2023) 106:249–56. doi: 10.1080/08164622.2022.2039554 35231199

[126] Liu J Zhao L Yan M Jin S Shang L Wang J . H4K79 and H4K91 histone lactylation, newly identified lactylation sites enriched in breast cancer. J Exp Clin Cancer Res. (2025) 44:252. doi: 10.1186/s13046-025-03512-6 40849487 PMC12374308

[127] Liu PK Chi YC Chang YC Lin YH Chen CY Liu C . Quercetin attenuates high glucose-induced VEGFA expression in ARPE-19 cells by inhibiting ROS generation, p38 MAPK phosphorylation, and NF-κB activation. Sci Rep. (2026) 16:4987. doi: 10.1038/s41598-026-35409-5 41520042 PMC12876867

[128] Du J Wang Y Tu Y Guo Y Sun X Xu X . A prodrug of epigallocatechin-3-gallate alleviates high glucose-induced pro-angiogenic factor production by inhibiting the ROS/TXNIP/NLRP3 inflammasome axis in retinal Müller cells. Exp Eye Res. (2020) 196:108065. doi: 10.1016/j.exer.2020.108065 32407725

[129] Zhang L Zhang ZK Liang S . Epigallocatechin-3-gallate protects retinal vascular endothelial cells from high glucose stress *in vitro* via the MAPK/ERK-VEGF pathway. Genet Mol Res. (2016) 15. doi: 10.4238/gmr.15027874 27323164

[130] Perdices L Fuentes-Broto L Segura F Cavero A Orduna-Hospital E Insa-Sánchez G . Systemic epigallocatechin gallate protects against retinal degeneration and hepatic oxidative stress in the P23H-1 rat. Neural Regener Res. (2022) 17:625–31. doi: 10.4103/1673-5374.320990 34380903 PMC8504391

[131] Lee HS Jun JH Jung EH Koo BA Kim YS . Epigalloccatechin-3-gallate inhibits ocular neovascularization and vascular permeability in human retinal pigment epithelial and human retinal microvascular endothelial cells via suppression of MMP-9 and VEGF activation. Molecules. (2014) 19:12150–72. doi: 10.3390/molecules190812150 25123184 PMC6270782

[132] Xu XH Zhao C Peng Q Xie P Liu QH . Kaempferol inhibited VEGF and PGF expression and *in vitro* angiogenesis of HRECs under diabetic-like environment. Braz J Med Biol Res. (2017) 50:e5396. doi: 10.1590/1414-431x20165396 28273207 PMC5378449

[133] Wu Y Zhang Q Zhang R . Kaempferol targets estrogen-related receptor α and suppresses the angiogenesis of human retinal endothelial cells under high glucose conditions. Exp Ther Med. (2017) 14:5576–82. doi: 10.3892/etm.2017.5261 29285095 PMC5740587

[134] Zhao L Sun J Shi S Qin X Zhang K Xu J . Kaempferol protects retinal ganglion ceils from high-glucose-induced injury by regulating vasohibin-1. Neurosci Lett. (2020) 716:134633. doi: 10.1016/j.neulet.2019.134633 31743752

[135] Albalawi FE Alsharif I Moawadh MS Alkhoshaiban A Falah Alshehri F Albalawi AE . Immunomodulatory effects of kaempferol on microglial and macrophage cells during the progression of diabetic retinopathy. Int Immunopharmacol. (2024) 133:112021. doi: 10.1016/j.intimp.2024.112021 38626549

[136] Zhang S Wang L Wu J Zhang Y . Deciphering the therapeutic mechanism of kaempferol in diabetic retinopathy via the P21/Thioredoxin axis. Mol Cell Biochem. (2026) 481:457–72. doi: 10.1007/s11010-025-05412-x 41129046 PMC12906542

[137] Pradhan G Kulkarni YA . Diabetes and its complications: Role of luteolin, a wonder chemical from the natural source. Curr Diabetes Rev. (2024) 21:e290224227537. doi: 10.2174/0115733998285798240217084632 38425118

[138] Yang Y Zhou M Liu H . Luteolin, an aryl hydrocarbon receptor antagonist, alleviates diabetic retinopathy by regulating the NLRP/NOX4 signalling pathway: Experimental and molecular docking study. Physiol Int. (2021) 108:172–84. doi: 10.1556/2060.2021.00148 34143751

[139] Zhang S Wu J Wang L Mu L Xu X Li J . SIRT1/P53 in retinal pigment epithelial cells in diabetic retinopathy: a gene co-expression analysis and He-Ying-Qing-Re formula treatment. Front Mol Biosci. (2024) 11:1366020. doi: 10.3389/fmolb.2024.1366020 38633216 PMC11021775

[140] Zhang S Wu J Zhang Y . Targeting mitophagy in diabetic retinopathy: novel insights into SQSTM1/BNIP3L pathway regulated by luteolin. Front Pharmacol. (2025) 16:1593213. doi: 10.3389/fphar.2025.1593213 40635752 PMC12237918

[141] Wang R Guo K Li J . Luteolin ameliorates diabetic retinopathy through activation of the Nrf2/Keap1 and SIRT3/AMPK antioxidant axes and suppression of cGAS-STING and Wnt/β-catenin pathways in experimental rats. J Physiol Biochem. (2026) 82. doi: 10.1007/s13105-026-01170-6 41793598

[142] Fu C Peng J Ling Y Zhao H Zhao Y Zhang X . Apigenin inhibits angiogenesis in retinal microvascular endothelial cells through regulating of the miR-140-5p/HDAC3-mediated PTEN/PI3K/AKT pathway. BMC Ophthalmol. (2023) 23:302. doi: 10.1186/s12886-023-03046-5 37415101 PMC10326941

[143] Li P Fang RL Wang W Zeng XX Lan T Liu SY . Apigenin suppresses epithelial-mesenchymal transition in high glucose-induced retinal pigment epithelial cell by inhibiting CBP/p300-mediated histone acetylation. Biochem Biophys Res Commun. (2024) 717:150061. doi: 10.1016/j.bbrc.2024.150061 38718570

[144] Yang L Li Z Fang J . Scutellarin alleviates diabetic retinopathy via the suppression of nucleotide-binding oligomerization domain (NOD)-like receptor pyrin domain containing protein 3 inflammasome activation. Curr Eye Res. (2024) 49:180–7. doi: 10.1080/02713683.2023.2273777 38014534

[145] Li N Guo XL Xu M Chen JL Wang YF Jie S . Network pharmacology mechanism of scutellarin to inhibit RGC pyroptosis in diabetic retinopathy. Sci Rep. (2023) 13:6504. doi: 10.1038/s41598-023-33665-3 37081038 PMC10119430

[146] Wang J Tan J Luo J Huang P Zhou W Chen L . Enhancement of scutellarin oral delivery efficacy by vitamin B12-modified amphiphilic chitosan derivatives to treat type II diabetes induced-retinopathy. J Nanobiotechnology. (2017) 15:18. doi: 10.1186/s12951-017-0251-z 28249594 PMC5333415

[147] Long L Li Y Yu S Li X Hu Y Long T . Scutellarin prevents angiogenesis in diabetic retinopathy by downregulating VEGF/ERK/FAK/Src pathway signaling. J Diabetes Res. (2019) 2019:4875421. doi: 10.1155/2019/4875421 31976335 PMC6949683

[148] Wang D Wang L Gu J Yang H Liu N Lin Y . Scutellarin inhibits high glucose-induced and hypoxia-mimetic agent-induced angiogenic effects in human retinal endothelial cells through reactive oxygen species/hypoxia-inducible factor-1α/vascular endothelial growth factor pathway. J Cardiovasc Pharmacol. (2014) 64:218–27. doi: 10.1097/fjc.0000000000000109 25192544

[149] Mei X Zhang T Ouyang H Lu B Wang Z Ji L . Scutellarin alleviates blood-retina-barrier oxidative stress injury initiated by activated microglia cells during the development of diabetic retinopathy. Biochem Pharmacol. (2019) 159:82–95. doi: 10.1016/j.bcp.2018.11.011 30447218

[150] Li W Xiao H . Dihydromyricetin alleviates high glucose-induced oxidative stress and apoptosis in human retinal pigment epithelial cells by downregulating miR-34a expression. Diabetes Metab Syndr Obes. (2021) 14:387–97. doi: 10.2147/dmso.S290633 33536772 PMC7850407

[151] Yang LP Sun HL Wu LM Guo XJ Dou HL Tso MO . Baicalein reduces inflammatory process in a rodent model of diabetic retinopathy. Invest Ophthalmol Vis Sci. (2009) 50:2319–27. doi: 10.1167/iovs.08-2642 19011009

[152] Liu C Fu Y Xu S Wang X Cao X . Uncovering the therapeutic mechanism of baicalein in diabetic retinopathy through ferroptosis regulation. Comput Methods Biomech BioMed Engin. (2026), 1–17. doi: 10.1080/10255842.2026.2646309 41848783

[153] Liao PL Lin CH Li CH Tsai CH Ho JD Chiou GC . Anti-inflammatory properties of shikonin contribute to improved early-stage diabetic retinopathy. Sci Rep. (2017) 7:44985. doi: 10.1038/srep44985 28322323 PMC5359562

[154] Oska N Awad AM Eltanani S Shawky M Naghdi A Yumnamcha T . Glyceraldehyde-3-phosphate dehydrogenase/1,3-bisphosphoglycerate-NADH as key determinants in controlling human retinal endothelial cellular functions: Insights from glycolytic screening. J Biol Chem. (2025) 301:108472. doi: 10.1016/j.jbc.2025.108472 40158853 PMC12136781

[155] Liu WY Liou SS Hong TY Liu IM . Hesperidin prevents high glucose-induced damage of retinal pigment epithelial cells. Planta Med. (2018) 84:1030–7. doi: 10.1055/a-0601-7020 29653456

[156] Liu WY Liou SS Hong TY Liu IM . Protective effects of hesperidin (citrus flavonone) on high glucose induced oxidative stress and apoptosis in a cellular model for diabetic retinopathy. Nutrients. (2017) 9. doi: 10.3390/nu9121312 29207476 PMC5748762

[157] Mao ZJ Liu SY Lan T Zeng XX Zhou LL Li P . Targeting DOT1L-mediated histone methylation by hesperetin alleviates retinal microvascular endothelial cell dysfunction and diabetic retinopathy. Biochem Pharmacol. (2025) 242:117402. doi: 10.1016/j.bcp.2025.117402 41047037

[158] Shehata AS Mohamed DA Hagras SM El-Beah SM Elnegris HM . The role of hesperidin in ameliorating retinal changes in rats with experimentally induced type 1 diabetes mellitus and the active role of vascular endothelial growth factor and glial fibrillary acidic protein. Anat Cell Biol. (2021) 54:465–78. doi: 10.5115/acb.21.105 34936987 PMC8693142

[159] Li R Du S Ye Z Yang W Liu Y . Blueberry anthocyanin extracts (BAEs) protect retinal and retinal pigment epithelium function from high-glucose-induced apoptosis by activating GLP-1R/Akt signaling. J Agric Food Chem. (2025) 73:5886–98. doi: 10.1021/acs.jafc.4c08978 40017023

[160] Kim J Kim KM Kim CS Sohn E Lee YM Jo K . Puerarin inhibits the retinal pericyte apoptosis induced by advanced glycation end products *in vitro* and *in vivo* by inhibiting NADPH oxidase-related oxidative stress. Free Radic Biol Med. (2012) 53:357–65. doi: 10.1016/j.freeradbiomed.2012.04.030 22609359

[161] Hao LN Wang M Ma JL Yang T . Puerarin decreases apoptosis of retinal pigment epithelial cells in diabetic rats by reducing peroxynitrite level and iNOS expression. Sheng Li Xue Bao. (2012) 64:199–206. doi: 10.1111/cpr.13153 22513471

[162] Sook Kim Y Soo Lee I Sook Kim J . Protective effects of puerariae radix extract and its single compounds on methylglyoxal-induced apoptosis in human retinal pigment epithelial cells. J Ethnopharmacol. (2014) 152:594–8. doi: 10.1016/j.jep.2014.01.008 24486213

[163] Zhu X Xie M Wang K Zhang K Gao Y Zhu L . The effect of puerarin against IL-1β-mediated leukostasis and apoptosis in retinal capillary endothelial cells (TR-iBRB2). Mol Vis. (2014) 20:1815–23. PMC4287719 25593509

[164] Cai Y Zhang X Xu X Yu Y . Effects of puerarin on the retina and STAT3 expression in diabetic rats. Exp Ther Med. (2017) 14:5480–4. doi: 10.3892/etm.2017.5203 29285079 PMC5740762

[165] Al-Dosari DI Ahmed MM Al-Rejaie SS Alhomida AS Ola MS . Flavonoid naringenin attenuates oxidative stress, apoptosis and improves neurotrophic effects in the diabetic rat retina. Nutrients. (2017) 9. doi: 10.3390/nu9101161 29064407 PMC5691777

[166] Xue B Wang Y . Naringenin upregulates GTPCH1/eNOS to ameliorate high glucose-induced retinal endothelial cell injury. Exp Ther Med. (2022) 23:428. doi: 10.3892/etm.2022.11355 35607381 PMC9121200

[167] Yuan D Xu Y Xue L Zhang W Gu L Liu Q . Resveratrol protects against diabetic retinal ganglion cell damage by activating the Nrf2 signaling pathway. Heliyon. (2024) 10:e30786. doi: 10.1016/j.heliyon.2024.e30786 38774075 PMC11107105

[168] Peng Y Hu L Xu H Fang J Zhong H . Resveratrol alleviates reactive oxygen species and inflammation in diabetic retinopathy via SIRT1/HMGB1 pathway-mediated ferroptosis. Toxicol Appl Pharmacol. (2025) 495:117214. doi: 10.1016/j.taap.2024.117214 39719253

[169] Wang Y Song SY Song Y Wang Y Wan ZW Sun P . Resveratrol protects Müller cells against ferroptosis in the early stage of diabetic retinopathy by regulating the Nrf2/GPx4/PTGS2 pathway. Mol Neurobiol. (2025) 62:3412–27. doi: 10.1007/s12035-024-04496-8 39292340

[170] Lin J Liang F Liu Y Shen Z Li X . HLA-A drives diabetic retinopathy pathogenesis: multi-omics integration reveals resveratrol's therapeutic potential via immunomodulation. J Transl Med. (2025) 23:1311. doi: 10.1186/s12967-025-07117-7 41254735 PMC12625036

[171] Wan ZW Sun P Song SY Wang Y Song Y Deng B . Resveratrol ameliorates early retinal neurodegeneration in diabetic retinopathy via microRNA-29b/specificity protein 1/apoptosis pathway by enhancing autophagy. Eur J Nutr. (2025) 64:232. doi: 10.1007/s00394-025-03751-5 40608078

[172] Li J Liu J Li J Tang Y Xiao Z Xu Y . Knocking down CDKN2A enhances the therapeutic effects of metformin and resveratrol on diabetic retinopathy. Appl Biochem Biotechnol. (2026). doi: 10.1007/s12010-026-05664-3 41843007

[173] Ran Z Zhang Y Wen X Ma J . Curcumin inhibits high glucose-induced inflammatory injury in human retinal pigment epithelial cells through the ROS-PI3K/AKT/mTOR signaling pathway. Mol Med Rep. (2019) 19:1024–31. doi: 10.3892/mmr.2018.9749 30569107 PMC6323224

[174] Bucolo C Drago F Maisto R Romano GL D'Agata V Maugeri G . Curcumin prevents high glucose damage in retinal pigment epithelial cells through ERK1/2-mediated activation of the Nrf2/HO-1 pathway. J Cell Physiol. (2019) 234:17295–304. doi: 10.1002/jcp.28347 30770549

[175] Wang L Sun X Zhu M Du J Xu J Qin X . Epigallocatechin-3-gallate stimulates autophagy and reduces apoptosis levels in retinal Müller cells under high-glucose conditions. Exp Cell Res. (2019) 380:149–58. doi: 10.1016/j.yexcr.2019.04.014 30998948

[176] Wang H Guan L Guan H Zhong J Zhong J . Gallic acid and vitamin C mitigate histopathological changes in the retina by attenuating dyslipidemia and mitigating oxidative stress, inflammation, and apoptosis in the eyes of type 2 diabetic rats induced with fructose/streptozotocin. Food Sci Nutr. (2025) 13:e70699. doi: 10.1002/fsn3.70699 40735405 PMC12305670

[177] Shanmuganathan S Angayarkanni N . Chebulagic acid chebulinic acid and gallic acid, the active principles of triphala, inhibit TNFα induced pro-angiogenic and pro-inflammatory activities in retinal capillary endothelial cells by inhibiting p38, ERK and NFkB phosphorylation. Vascul Pharmacol. (2018) 108:23–35. doi: 10.1016/j.vph.2018.04.005 29678603

[178] Nair VR RV G Sr. RP P . Aldose reductase and protein glycation inhibitory activity of dark chocolate-assisted zinc oxide nanoparticles. Cureus. (2023) 15:e48953. doi: 10.7759/cureus.48953 38111407 PMC10726068

[179] Xu Z Li S Li K Wang X Li X An M . Urolithin A ameliorates diabetic retinopathy via activation of the Nrf2/HO-1 pathway. Endocr J. (2022) 69:971–82. doi: 10.1507/endocrj.EJ21-0490 35321989

[180] Raghu G Akileshwari C Reddy VS Reddy GB . Attenuation of diabetic retinopathy in rats by ellagic acid through inhibition of AGE formation. J Food Sci Technol. (2017) 54:2411–21. doi: 10.1007/s13197-017-2683-8 28740299 PMC5502036

[181] Ouyang H Xie Y Du A Dong S Zhou S Lu B . Chlorogenic acid ameliorates non-proliferative diabetic retinopathy via alleviating retinal inflammation through targeting TNFR1 in retinal endothelial cells. Int Immunopharmacol. (2024) 141:112929. doi: 10.1016/j.intimp.2024.112929 39153307

[182] Vieira LC Moreira CPS Castro BFM Cotta OAL Silva LM Fulgêncio GO . Rosmarinic acid intravitreal implants: A new therapeutic approach for ocular neovascularization. Planta Med. (2020) 86:1286–97. doi: 10.1055/a-1223-2525 32797466

[183] Scripsema NK Hu DN Rosen RB . Lutein, zeaxanthin, and meso-zeaxanthin in the clinical management of eye disease. J Ophthalmol. (2015) 2015:865179. doi: 10.1155/2015/865179 26819755 PMC4706936

[184] Li ZZ Lu XZ Ma CC Chen L . Serum lycopene levels in patients with diabetic retinopathy. Eur J Ophthalmol. (2010) 20:719–23. doi: 10.1177/112067211002000412 20099237

[185] Xiang XH Wei J Wang XF Xu Q Yu CL He CL . Lychee seed polyphenol ameliorates DR via inhibiting inflammasome/apoptosis and angiogenesis in hRECs and db/db mice. BioMed Pharmacother. (2023) 167:115478. doi: 10.1016/j.biopha.2023.115478 37703661

[186] Paramaswaran Y Subramanian A Paramakrishnan N Ramesh M Muthuraman A . Therapeutic investigation of palm oil mill effluent-derived beta-carotene in streptozotocin-induced diabetic retinopathy via the regulation of blood-retina barrier functions. Pharm (Basel). (2023) 16. doi: 10.3390/ph16050647 37242430 PMC10224388

[187] Benlarbi-Ben Khedher M Hajri K Dellaa A Baccouche B Hammoum I Boudhrioua-Mihoubi N . Astaxanthin inhibits aldose reductase activity in Psammomys obesus, a model of type 2 diabetes and diabetic retinopathy. Food Sci Nutr. (2019) 7:3979–85. doi: 10.1002/fsn3.1259 31890176 PMC6924305

[188] Baccouche B Benlarbi M Barber AJ Ben Chaouacha-Chekir R . Short-term administration of astaxanthin attenuates retinal changes in diet-induced diabetic Psammomys obesus. Curr Eye Res. (2018) 43:1177–89. doi: 10.1080/02713683.2018.1484143 30028214

[189] Janani R Anitha RE Perumal MK Divya P Baskaran V . Astaxanthin mediated regulation of VEGF through HIF1α and XBP1 signaling pathway: An insight from ARPE-19 cell and streptozotocin mediated diabetic rat model. Exp Eye Res. (2021) 206:108555. doi: 10.1016/j.exer.2021.108555 33789142

[190] Yin Z Tan R Yuan T Chen S Quan Y Hao Q . Berberine prevents diabetic retinopathy through inhibiting HIF-1α /VEGF/ NF-κ B pathway in db/db mice. Pharmazie. (2021) 76:165–71. doi: 10.1691/ph.2021.01012 33849702

[191] Fang W Huang X Wu K Zong Y Yu J Xu H . Activation of the GABA-alpha receptor by berberine rescues retinal ganglion cells to attenuate experimental diabetic retinopathy. Front Mol Neurosci. (2022) 15:930599. doi: 10.3389/fnmol.2022.930599 36017075 PMC9396352

[192] Wang N Zhang C Xu Y Tan HY Chen H Feng Y . Berberine improves insulin-induced diabetic retinopathy through exclusively suppressing Akt/mTOR-mediated HIF-1α/VEGF activation in retina endothelial cells. Int J Biol Sci. (2021) 17:4316–26. doi: 10.7150/ijbs.62868 34803500 PMC8579442

[193] Li N Chen JL Sun YJ Sun JF Wang TH Saik AYH . Berberine attenuates the expression of NLRP3 and downstream inflammasome effectors in diabetic retinopathy. J Nat Med. (2025) 79:1091–105. doi: 10.1007/s11418-025-01935-1 40779223

[194] Chen H Ji Y Yan X Su G Chen L Xiao J . Berberine attenuates apoptosis in rat retinal Müller cells stimulated with high glucose via enhancing autophagy and the AMPK/mTOR signaling. BioMed Pharmacother. (2018) 108:1201–7. doi: 10.1016/j.biopha.2018.09.140 30372821

[195] Ai X Yu P Luo L Sun J Tao H Wang X . Berberis dictyophylla F. inhibits angiogenesis and apoptosis of diabetic retinopathy via suppressing HIF-1α/VEGF/DLL-4/Notch-1 pathway. J Ethnopharmacol. (2022) 296:115453. doi: 10.1016/j.jep.2022.115453 35697191

[196] Tang L Zhang C Yang Q Xie H Liu D Tian H . Melatonin maintains inner blood-retinal barrier via inhibition of p38/TXNIP/NF-κB pathway in diabetic retinopathy. J Cell Physiol. (2021) 236:5848–64. doi: 10.1002/jcp.30269 33432588

[197] Obrosova IG Stevens MJ Lang HJ . Diabetes-induced changes in retinal NAD-redox status: pharmacological modulation and implications for pathogenesis of diabetic retinopathy. Pharmacology. (2001) 62:172–80. doi: 10.1159/000056091 11287819

[198] Kim YS Kim M Choi MY Lee DH Roh GS Kim HJ . Alpha-lipoic acid reduces retinal cell death in diabetic mice. Biochem Biophys Res Commun. (2018) 503:1307–14. doi: 10.1016/j.bbrc.2018.07.041 30017190

[199] Chen F Yang X Yin Y Meng Z Wu Z Liu D . Melatonin alleviates retina angiogenesis by targeting fibronectin and the VEGF pathway. FASEB J. (2025) 39:e71073. doi: 10.1096/fj.202500814RR 41026035

[200] Ning J Pan M Yang H Wang Z Wang X Guo K . Melatonin attenuates diabetic retinopathy by regulating EndMT of retinal vascular endothelial cells via inhibiting the HDAC7/FOXO1/ZEB1 axis. J Pineal Res. (2024) 76:e13008. doi: 10.1111/jpi.13008 39300782

[201] Tang L Zhang C Lu L Tian H Liu K Luo D . Melatonin maintains inner blood-retinal barrier by regulating microglia via inhibition of PI3K/Akt/Stat3/NF-κB signaling pathways in experimental diabetic retinopathy. Front Immunol. (2022) 13:831660. doi: 10.3389/fimmu.2022.831660 35371022 PMC8964465

[202] Tu Y Zhu M Wang Z Wang K Chen L Liu W . Melatonin inhibits Müller cell activation and pro-inflammatory cytokine production via upregulating the MEG3/miR-204/Sirt1 axis in experimental diabetic retinopathy. J Cell Physiol. (2020) 235:8724–35. doi: 10.1002/jcp.29716 32324260

[203] Yu Z Lu B Sheng Y Zhou L Ji L Wang Z . Andrographolide ameliorates diabetic retinopathy by inhibiting retinal angiogenesis and inflammation. Biochim Biophys Acta. (2015) 1850:824–31. doi: 10.1016/j.bbagen.2015.01.014 25641276

[204] Kumar MP Mamidala E Al-Ghanim KA Al-Misned F Mahboob S . Evaluation of the andrographolides role and its indoleamine 2,3-dioxygenase inhibitory potential and attendant molecular mechanism against STZ-induced diabetic rats. Saudi J Biol Sci. (2020) 27:713–9. doi: 10.1016/j.sjbs.2019.12.007 32210693 PMC6997866

[205] Guo ZL Li Y Liu XW Wu MY Guo Q Yao XC . Sodium tanshinone IIA silate alleviates high glucose induced barrier impairment of human retinal pigment epithelium through the reduction of NF-κB activation via the AMPK/p300 pathway. Curr Eye Res. (2020) 45:177–83. doi: 10.1080/02713683.2019.1668419 31576759

[206] Zeng X Deng Y Yuan M He Q Wu Y Li S . Study on the antioxidant effect of tanshinone IIA on diabetic retinopathy and its mechanism based on integrated pharmacology. Evid Based Complement Alternat Med. (2022) 2022:9990937. doi: 10.1155/2022/9990937 36437835 PMC9691304

[207] Dong S Zhang Y Xie Y Ouyang H Zhou S Shi J . Uncovering the potential mechanism and bioactive compounds of Salviae miltiorrhizae radix et rhizoma in attenuating diabetic retinopathy. Phytomedicine. (2025) 139:156461. doi: 10.1016/j.phymed.2025.156461 39986223

[208] Fang J Chang X . Celastrol inhibits the proliferation and angiogenesis of high glucose-induced human retinal endothelial cells. BioMed Eng Online. (2021) 20:65. doi: 10.1186/s12938-021-00904-5 34193168 PMC8244207

[209] Yang H Gan S Jiang Z Song X Chen T Xu Y . Protective effects of essential oil from Fructus Alpiniae zerumbet on retinal Müller gliosis via the PPAR-γ-p-CREB signaling pathway. Chin Med. (2020) 15:4. doi: 10.1186/s13020-019-0283-4 31938037 PMC6954544

[210] Zhu D Zou W Cao X Xu W Lu Z Zhu Y . Ferulic acid attenuates high glucose-induced apoptosis in retinal pigment epithelium cells and protects retina in db/db mice. PeerJ. (2022) 10:e13375. doi: 10.7717/peerj.13375 35669949 PMC9165606

[211] Khatun MM Bhuia MS Chowdhury R Sheikh S Ajmee A Mollah F . Potential utilization of ferulic acid and its derivatives in the management of metabolic diseases and disorders: An insight into mechanisms. Cell Signal. (2024) 121:111291. doi: 10.1016/j.cellsig.2024.111291 38986730

[212] Sun HQ Zhou ZY . Effect of ginsenoside-Rg3 on the expression of VEGF and TNF-α in retina with diabetic rats. Int J Ophthalmol. (2010) 3:220–3. doi: 10.3980/j.issn.2222-3959.2010.03.09 22553558 PMC3340625

[213] Xue LP Fu XL Hu M Zhang LW Li YD Peng YL . Rg1 inhibits high glucose-induced mesenchymal activation and fibrosis via regulating miR-2113/RP11-982M15.8/Zeb1 pathway. Biochem Biophys Res Commun. (2018) 501:827–32. doi: 10.1016/j.bbrc.2018.04.055 29654764

[214] Ying Y Zhang YL Ma CJ Li MQ Tang CY Yang YF . Neuroprotective effects of ginsenoside Rg1 against hyperphosphorylated tau-induced diabetic retinal neurodegeneration via activation of IRS-1/Akt/GSK3β signaling. J Agric Food Chem. (2019) 67:8348–60. doi: 10.1021/acs.jafc.9b02954 31304751

[215] Tang K Qin W Wei R Jiang Y Fan L Wang Z . Ginsenoside Rd ameliorates high glucose-induced retinal endothelial injury through AMPK-STRT1 interdependence. Pharmacol Res. (2022) 179:106123. doi: 10.1016/j.phrs.2022.106123 35150861

[216] Li B Zhang DC Li XW Dong XN Li WP Li WZ . Protective effect of ginsenoside Rg_1 aganist diabetic retinopathy by inhibiting NLRP3 inflammasome in type 2 diabetic mice. Zhongguo Zhong Yao Za Zhi. (2022) 47:476–83. doi: 10.19540/j.cnki.cjcmm.20210926.401 35178992

[217] Xue L Hu M Li J Li Y Zhu Q Zhou G . Ginsenoside Rg1 inhibits high glucose-induced proliferation, migration, and angiogenesis in retinal endothelial cells by regulating the lncRNA SNHG7/miR-2116-5p/SIRT3 axis. J Oncol. (2022) 2022:6184631. doi: 10.1155/2022/6184631 36510610 PMC9741534

[218] Xue L Hu M Zhu Q Li Y Zhou G Zhang X . GRg1 inhibits the TLR4/NF-κB signaling pathway by upregulating miR-216a-5p to reduce growth factors and inflammatory cytokines in DR. Mol Biol Rep. (2023) 50:9379–94. doi: 10.1007/s11033-023-08895-3 37819496 PMC10635910

[219] Hu RY Qi SM Wang YJ Li WL Zou WC Wang Z . Ginsenoside Rg3 improved age-related macular degeneration through inhibiting ROS-mediated mitochondrion-dependent apoptosis *in vivo* and *in vitro*. Int J Mol Sci. (2024) 25. doi: 10.3390/ijms252111414 39518966 PMC11547035

[220] Liu J Zhang Y Xu X Dong X Pan Y Sun X . Ginsenoside Ro prevents endothelial injury via promoting Epac1/AMPK- mediated mitochondria protection in early diabetic retinopathy. Pharmacol Res. (2025) 211:107562. doi: 10.1016/j.phrs.2024.107562 39732351

[221] Xue L Hu M Li Y Zhu Q Zhou G Zhang X . Ginsenoside Rg1 inhibits angiogenesis in diabetic retinopathy through the miR-100-3p/FBXW7/c-MYC molecular axis. J Diabetes Investig. (2025) 16:791–806. doi: 10.1111/jdi.70016 40033576 PMC12057387

[222] Chen Y Jin Y . Ginsenoside Rg1 alleviates HG-induced autophagy of retinal microvascular endothelial cells by activating ACE2/Ang1-7/Mas *in vitro*. Sci Rep. (2025) 15:45315. doi: 10.1038/s41598-025-29344-0 41290961 PMC12749397

[223] Harley O Amelia YS Gustianty E Soetedjo NNM Kartasasmita AS . Resveratrol as a multitarget modulator in diabetic retinopathy: a systematic review of *in vitro* and *in vivo* studies. BMC Ophthalmol. (2026) 26:55. doi: 10.1186/s12886-026-04623-0 41606717 PMC12853864

[224] Chen Q Qiu FS Xie W Yu WY Su ZA Qin GM . Gypenoside A-loaded mPEG-PLGA nanoparticles ameliorate high-glucose-induced retinal microvasculopathy by inhibiting ferroptosis. Int J Pharm. (2024) 666:124758. doi: 10.1016/j.ijpharm.2024.124758 39326476

[225] Luo Y Dong X Lu S Gao Y Sun G Sun X . Gypenoside XVII alleviates early diabetic retinopathy by regulating Müller cell apoptosis and autophagy in db/db mice. Eur J Pharmacol. (2021) 895:173893. doi: 10.1016/j.ejphar.2021.173893 33493483

[226] Dongare S Gupta SK Mathur R Saxena R Mathur S Agarwal R . Zingiber officinale attenuates retinal microvascular changes in diabetic rats via anti-inflammatory and antiangiogenic mechanisms. Mol Vis. (2016) 22:599–609. PMC4898299 27293376

[227] Li H Li R Wang L Liao D Zhang W Wang J . Proanthocyanidins attenuate the high glucose-induced damage of retinal pigment epithelial cells by attenuating oxidative stress and inhibiting activation of the NLRP3 inflammasome. J Biochem Mol Toxicol. (2021) 35:e22845. doi: 10.1002/jbt.22845 34338401

[228] Zuo L Ye M Chen L Xu X Huang S Li X . Anthocyanin-rich berries and their bioactive compounds in ocular health: Mechanisms and therapeutic potential. J Agric Food Chem. (2025) 73:23724–41. doi: 10.1021/acs.jafc.5c03411 40956067

[229] Khosravifar M Sajadimajd S Bahrami G . Anti-diabetic effects of macronutrients via modulation of angiogenesis: A comprehensive review on carbohydrates and proteins. Curr Mol Med. (2023) 23:250–65. doi: 10.2174/1566524022666220321125548 35319367

[230] Zucca G Vigani B Valentino C Ruggeri M Marchesi N Pascale A . Chondroitin sulphate-chitosan based nanogels loaded with naringenin-β-cyclodextrin complex as potential tool for the treatment of diabetic retinopathy: A formulation study. Int J Nanomedicine. (2025) 20:907–32. doi: 10.2147/ijn.S488507 39867306 PMC11766310

[231] Gonzalez-Perez J Lopera-Echavarría AM Arevalo-Alquichire S Araque-Marín P Londoño ME . Development of a resveratrol nanoformulation for the treatment of diabetic retinopathy. Materials (Basel). (2024) 17. doi: 10.3390/ma17061420 38541574 PMC10972098

[232] Yu X Wang X . Formulation of a novel drug by the Olea europaea green-formulated silver nanoparticles for the treatment of diabetic retinopathy. Appl Organomet Chem. (2025) 39:e70207. doi: 10.1002/aoc.7020742587375

[233] Jin Y Cai T Huang J Chen L Lin Y Liu J . Suppression of retinal neovascularisation by tetrahedral framework nucleic acids-resveratrol via dual anti-angiogenesis and anti-inflammation. Cell Prolif. (2026):e70227. doi: 10.1111/cpr.70227 42233589 PMC13325993

